# Flow of online misinformation during the peak of the COVID-19 pandemic in Italy

**DOI:** 10.1140/epjds/s13688-021-00289-4

**Published:** 2021-07-06

**Authors:** Guido Caldarelli, Rocco De Nicola, Marinella Petrocchi, Manuel Pratelli, Fabio Saracco

**Affiliations:** 1 Department of Molecular Sciences and Nanosystems, Ca’Foscari University of Venice, Ed. Alfa, Via Torino 155, 30170 Venezia Mestre, Italy; 2 European Centre for Living Technology (ECLT), Ca’ Bottacin, 3911 Dorsoduro Calle Crosera, 30123 Venice, Italy; 3 IMT School For Advanced Studies Lucca, Piazza San Francesco 19, 55100 Lucca, Italy; 4 Institute of Informatics and Telematics, National Research Council, via Moruzzi 1, 56124 Pisa, Italy; 5 CINI – National Laboratory for Cybersecurity, via Ariosto, 25, 00185 Roma, Italy

**Keywords:** COVID-19 Infodemic, Misinformation, Twitter

## Abstract

The COVID-19 pandemic has impacted on every human activity and, because of the urgency of finding the proper responses to such an unprecedented emergency, it generated a diffused societal debate. The online version of this discussion was not exempted by the presence of misinformation campaigns, but, differently from what already witnessed in other debates, the COVID-19 -intentional or not- flow of false information put at severe risk the public health, possibly reducing the efficacy of government countermeasures. In this manuscript, we study the *effective* impact of misinformation in the Italian societal debate on Twitter during the pandemic, focusing on the various discursive communities. In order to extract such communities, we start by focusing on verified users, i.e., accounts whose identity is officially certified by Twitter. We start by considering each couple of verified users and count how many unverified ones interacted with both of them via tweets or retweets: if this number is statically significant, i.e. so great that it cannot be explained only by their activity on the online social network, we can consider the two verified accounts as similar and put a link connecting them in a monopartite network of verified users. The discursive communities can then be found by running a community detection algorithm on this network.

We observe that, despite being a mostly scientific subject, the COVID-19 discussion shows a clear division in what results to be different political groups. We filter the network of retweets from random noise and check the presence of messages displaying URLs. By using the well known browser extension NewsGuard, we assess the trustworthiness of the most recurrent news sites, among those tweeted by the political groups. The impact of low reputable posts reaches the 22.1% in the right and center-right wing community and its contribution is even stronger in absolute numbers, due to the activity of this group: 96% of all non reputable URLs shared by political groups come from this community.

**Supplementary Information:**

The online version contains supplementary material available at 10.1140/epjds/s13688-021-00289-4.

## Introduction

The advent of the internet and online social media has promoted a more democratic access to information, increasing the offer of news sources, with a significant number of individual contributions too. Unfortunately, unmediated communication channels have generated an incredible amount of low-quality contents, polluting the online debate in several areas, like politics, healthcare, education, and environment [1].

For this reason, in the recent Joint Communication titled “Tackling COVID-19 disinformation – Getting the facts right” (June 10, 2020, https://bit.ly/35C1dGs), the High Representative of the Union for Foreign Affairs and Security Policy, while introducing the various d/misinformation campaigns that arose during the first months of the COVID-19 pandemic, presented an explicit declaration of intent: “Combating the flow of disinformation, misinformation […] calls for action through the EU’s existing tools, as well as with Member States’ competent authorities […] enhancing citizens’ resilience.”

Detecting misinformation campaigns and investigating pollution in online political debates have been the target of many studies, see, e.g., [2–11]. Nevertheless, the analysis of the existence and diffusion of polarised/biased/false stories about COVID-19 has immediately attracted several scholars, which are focusing on different facets of these phenomena, such as: the most searched terms on Google related to COVID-19 [12], the existence of Facebook groups experiencing an extreme exposure to disinformation [13], the change in the type of information on Twitter during the evolution of the pandemic [14] and the *disinformation epidemiology* on various online social platforms [15]. In the present paper, using Twitter as a benchmark, we shall consider the *effective* flow of online misinformation in Italy, one of the countries in Europe that have been affected the most by COVID-19 in Spring, 2020,^1^ and how this flow affected the various discursive communities, i.e., groups of users that debate on the pandemic. Since the debate is mostly centered on verified users, i.e., users whose identity is certified by Twitter, we start considering their interactions with unverified accounts. Following [10, 11, 16], our intuition is that two verified users, perceived as similar by unverified users, interact with (i.e., retweet and are retweeted by) the same accounts. In order to assess how many common unverified users are ‘enough’ to state that two verified users are indeed similar, we use an entropy-based null-model as a benchmark [17, 18]. In a nutshell, the entropy-based null-model is a network benchmark in which part of the information is constrained to the values observed in the real system and the rest is completely random. If the observations are not compatible with the null-model, then they cannot be explained by the constraints only and carry a non trivial information regarding the real system.

Interestingly enough, we find that the main discursive communities are political, i.e., they involve politicians, political parties and journalists supporting a specific political ideal. While, at first sight, this may sound surprising – the pandemic debate was more on a scientific than on a political ground, at least in the very first phase of its abrupt diffusion –, it might be due to pre-existing *echo chambers* [19].

We then consider the news sources shared among the accounts of the various groups. Through a hybrid annotation approach, based on the judgments of independent journalists and annotation carried out by members of our team, we categorise such sources as reputable or not (in terms of credibility of published news and transparency of editorial policies).

Finally, we extract the effective flow of content shared within the network: still following the approach of Ref. [10, 11], we extend the entropy-based methodology to a directed bipartite network of users and posts. In this sense, we are able to control not only the authorship activity and the retweeting attitude of the various accounts, but even the *virality* of the different messages, i.e., how many times a single message is shared.

The various political groups display different online behaviours. In particular, the right wing community is more numerous and more active, even relatively to the number of accounts involved, than the other communities. Surprisingly enough, newly formed political parties, as the one of the former Italian Prime Minister Matteo Renzi, quickly imposed their presence on Twitter with a strong activity. Furthermore, the different political parties use different sources for getting information on the spreading on the pandemic. Notably, we experience that right and center-right wing accounts spread information from non reputable sources with a frequency almost 10 times higher than that of the other political groups. Due to their outstanding activity, their impact, in terms of number of d/misinforming posts in the debate, is much greater than that of any other group.

The paper is organised as follows: Sect. 2 presents related work on the analysis of low-credible information regarding the pandemic. In Sect. 3, we introduce our dataset, while the results of our analysis are given in Sect. 4. After discussing the results in Sect. 5, we introduce the methodology in Sect. 6.

## Related work

As in any disaster, natural or otherwise, people are exposed to online misinformation. This is the case of COVID-19 too: the physical pandemic was quickly complemented by the so-called COVID-19 infodemic, i.e., the diffusion of a great amount of low-quality information about the virus. Academia has stepped up its efforts to combat this infodemic. Here, we briefly review some of the most relevant articles in the area.

Rovetta et al., in [12], explore the internet search activity related to COVID-19 from January to March 2020, to analyse article titles from the most read newspapers and government websites, ‘to investigate the attitudes of infodemic monikers circulating across various regions and cities in Italy’. The study reveals a growing regional and population-level interest in COVID-19 in Italy, highlighting how the majority of searches concern – often unfounded – remedies against the disease.

Work in [14], by Gallotti et al., develops an Infodemic Risk Index to depict the risk of exposure to false information in various countries around the world. Regarding healthcare news, the authors find that even before the rise of the pandemic, entire countries were exposed to false stories that can severely threaten public health.

Hossaini et al. [20] release COVIDLies, a dataset of 6761 expert-annotated tweets to evaluate the performances of existing NLP systems in detecting false stories about COVID-19. Still regarding datasets, work by Zhou et al. [21] presents ReCOVery, a repository of more than 2k news articles on Coronavirus, together with more than 140k tweets testifying the spreading of such articles on Twitter. Chen et al., in [22], present to the scientific community a multilingual COVID-19 Twitter dataset that they have been continuously collecting since January 2020. Celestini et al., in [13], collect and analyse over 1.5 M COVID-19-related posts in Italian. Findings are that, although controversial topics associated to the origin of the virus circulate on social networks, discussions on such topics is negligible compared to those on mainstream news websites.

Pierri et al., in [23], provide public access to online conversations of Italian users around vaccines on Twitter. This represents an on-going collection capturing the Italian vaccine roll-out (on December 27, 2020). The authors report a consistent amount of low-credibility information already circulating on Twitter alongside vaccine-related conversations. Still regarding COVID-19 vaccination campaigns, De Verna et al. collect a Twitter dataset of English posts, giving statistics about hashtags, URLs, and number of tweets over time, through a dashboard.

Sharma et al, in [24], consider the role of Twitter bots in the pandemic online debate. By moving away from the research trend of detecting bot squads, on the basis of features concerning coordination and synchronous behavior among a group of accounts, they propose an approach to automatically uncover coordinated group behaviours from account activities and interactions between accounts, based on temporal point processes.

A lot of work examines Twitter, because of the availability of public APIs for data gathering. Instead, Yang et al. [25] analyse and compare the presence of links pointing to low-credibility content both on Twitter and Facebook. Misinformation ‘superspreaders’ and evidences of coordinated sharing of false stories about COVID-19 are present on both the platforms. Still at a narrower granularity, Cinelli et al., in [15], carry on a massive analysis on Twitter, Instagram, YouTube, Reddit and Gab. The authors characterize COVID-19 information spreading from questionable sources, finding different volumes of misinformation in each platform.

This brief literature overview on the COVID-19 infodemic, although not exhaustive, highlights that the spread of misinformation on pandemic-related issues on the internet and social media is a major issue. Scientists propose various methods to detect false information about the virus. Aligned with this line of research, in this manuscript we quantify the *effective* level of misinformation about the pandemic exchanged on Twitter during late winter and early spring in 2020 in Italy, with a special focus on the role of the Italian political communities.

## Dataset

Using the Twitter’s streaming API from February 21st to April 20th 2020, we collected circa 4.5M tweets in Italian.^2^ Actually, the dataset analysed is a subset of a greater corpus, in which the language was not a selection criterion for the download; thus, we than selected Italian messages only. Also, due to the great amount of data to potentially download, we experimented some loss of information, due to the APIs rate limits, even if quite seldom;^3^ however, due to the validation procedure we applied to our network, we expect the impact of the Twitter rate limits to be negligible, for the results evaluation.

The data collection was keyword- and hashtag-based and related to COVID-19 pandemic; the complete list of keywords and hashtags used for the data collection can be found in Table 1. Let us remind that the Twitter’s streaming API returns any tweet containing those terms in the text of the tweet, as well as in its metadata. It is worth noting that it is not always necessary to have each permutation of a specific keyword in the tracking list. For example, the keyword ‘COVID’ would return tweets that contain also both ‘COVID19’ and ‘COVID-19’.

**Table 1:** Keywords and Hashtags which drove the data collection phase

Keywords and Hashtags
coronavirus
ncov
covid
SARS-CoV2
#coronavirus
#coronaviruses
#WuhanCoronavirus
#CoronavirusOutbreak
#coronaviruschina
#coronaviruswuhan
#ChinaCoronaVirus
#nCoV
#ChinaWuHan
#nCoV2020
#nCov2019
#covid2019
#covid-19
#SARS_CoV_2
#SARSCoV2
#COVID19

We would like to remark that, though less popular in Italy than other social platforms (statistics say that Twitter is used by less than 5.8% of the Italian population [26]), its usage by journalists and politicians is higher than other platforms. Specifically, Twitter is the second most used social platform, after Facebook, with an incidence of 30% of journalists accessing it every day [27]. This is probably due to the limited number of characters allowed in tweets, which is extremely suitable for short and fast communication, as the breaking news.

Finally, details about the health situation in Italy during the period of data collection can be found in the Additional file 1, Sect. 1.1: ‘Evolution of the COVID-19 pandemic in Italy’.

## Results

### Discursive communities of verified users

Many studies in the field of social network analysis show that users are highly clustered per similar opinions [28–36]. Following the example of references [10, 11], we leverage this users’ clustering in order to detect discursive communities, i.e., account groups interacting between each other by retweeting on the same (Covid-related) subjects. Remarkably, our methodology does not consider the shared texts, being focused on the retweeting activity among users only. Here, we will examine how the information about discursive communities of verified Twitter users can be extracted.

On Twitter, there are two distinct categories of accounts: verified and unverified users. The former are usually owned by politicians, journalists or VIPs in general, as well as ministers, newspapers, newscasts, companies, and so on: for that kind of users, the verification procedure guarantees the identity of their accounts. Although the identity of verified accounts is certified, their content cannot be considered reliable a priori (just as in the case of unverified accounts). However, the information carried by verified accounts has been studied extensively in order to have a sort of anchor for the related discussion [9–11, 16, 37, 38]

To detect the discursive communities, we consider the bipartite network represented by verified (on one layer) and unverified (on the other layer) accounts: a link connects the verified user *v* with the unverified one *u* if *v* is retweeted by *u* at least once, and/or viceversa. To extract the similarity of users, we compare the observed commonalities with those expected by a bipartite entropy-based null-model, the Bipartite Configuration Model (*BiCM* [39]), described in details in Sect. 6.1. The rationale is that two verified users, connected to the same unverified accounts, have similar visions, as perceived by the audience represented by unverified accounts. We thus apply the method of [40], in order to get a statistically validated projection of the bipartite network of verified and unverified users. In a nutshell, the idea is to compare the amount of common linkage measured on the real network with the expectations of an entropy-based null-model fixing (on average) the degree sequence: if the associated p-value is statistically significant, i.e. it is so low that the measurement cannot be explained by the model, it carries non trivial information. We then build an undirected monopartite (validated) projection of verified users in which two nodes are connected if their p-value is statistically significant.

**Figure 1:**
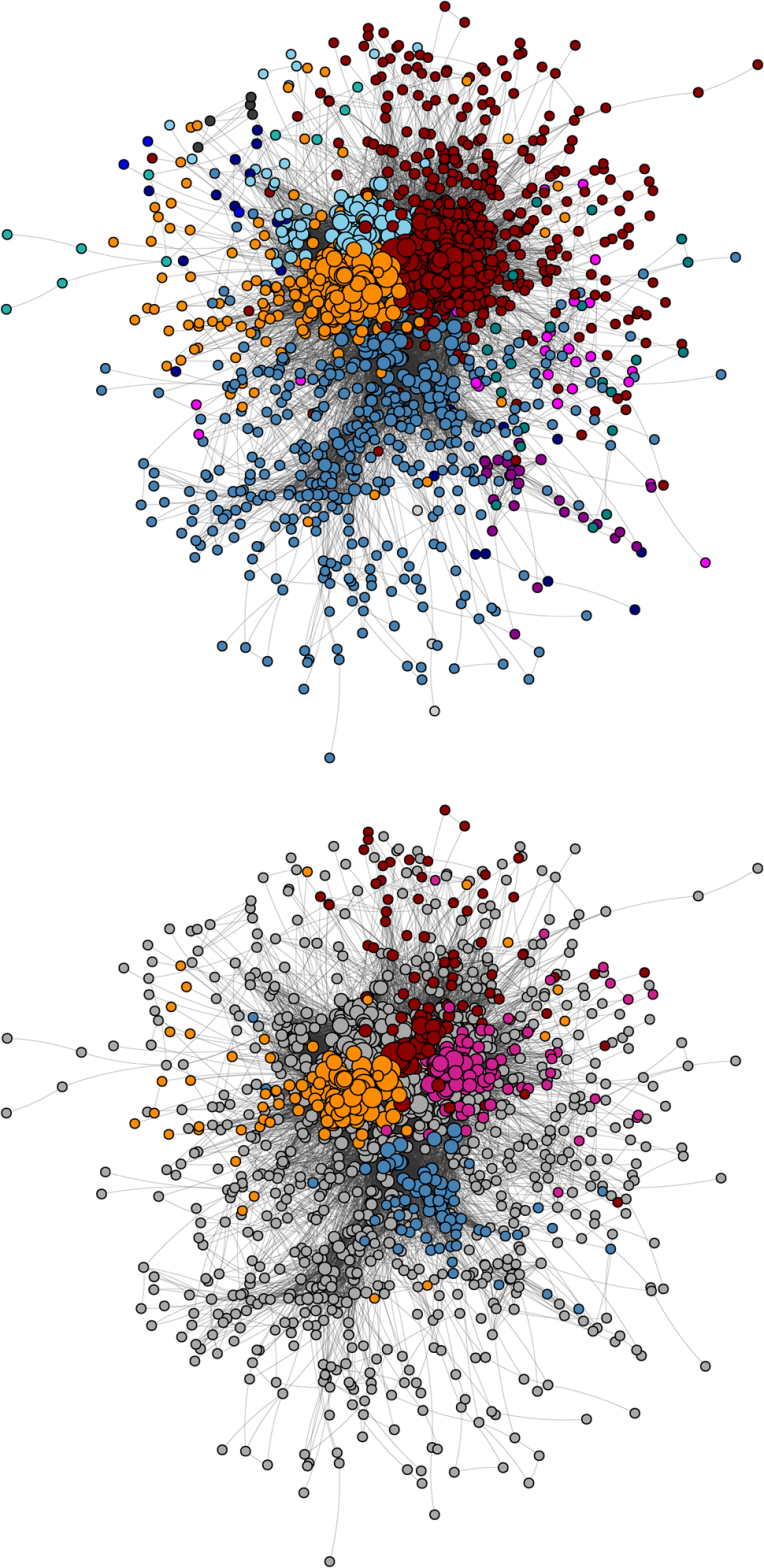
Discursive communities of verified users. They have been found running the Louvain community detection algorithm on the Largest Connected Component (LCC) of the validated network of verified users. Top panel: In red, top right corner, there are the center-left wing parties; in sky blue (on top), there are the official government accounts; in orange, the M5S-oriented community and in steel blue (on the bottom) the news media and center-right and right wing communities. Other minor communities can be found in the periphery of the LCC. Actually, by rerunning the same community detection algorithm inside these larger communities, it is possible to find *purely* political subcommunities, i.e., communities composed quite exclusively by politicians and official accounts of political parties. This can be seen in the lowest panel: in magenta, Italia Viva, the political party of the former Prime Minister Matteo Renzi; in red, the Partito Democratico, i.e., the Italian Democratic Party; in orange, M5S and in blue the center-right and right wing parties Forza Italia, Lega and Fratelli d’Italia. A more detailed description of the subcommunities of the network can be found in Sect. 2 of the Additional file 1. In both panels, the node dimensions are proportional to their degree. The layout used for network visualization is the Fruchterman-Reingold one [43]

The top panel of Fig. 1 shows the network obtained by following the above procedure. Hereafter, a network resulting from the projection procedure will be called *validated* network.^4^

In order to get the community of verified users, we applied the Louvain algorithm [41] to the data in the undirected validated network. Such an algorithm, despite being one of the most effective and popular, is also known to be order dependent [42]. To get rid of this bias, we apply it iteratively *N* times (*N* being the number of the nodes), after reshuffling the order of the nodes. Finally, we select the partition with the highest modularity. The network presents a strong community structure, composed by four main subgraphs. When analysing them, we find that they correspond to

1. Media and right/center-right wing parties (in steel blue);
2. Center-left wing (in dark red);
3. Movimento 5 Stelle (*5 Stars Movement*, or M5S; in dark orange);
4. Institutional accounts (in sky blue).

Details about the political situation in Italy during the period of data collection can be found in the Additional file 1, Sect. 1.2: ‘Italian political situation during the COVID-19 pandemic’.

While the various groups display a quite evident homophily among their elements, we further examined them by re-running the Louvain algorithm inside each of them, with the same care as above for the node order.

Since the subcommunities structure is extremely rich, we invite the interested reader to consult Sect. 2 of the Additional file 1 for a more detailed description. Hereafter, we will focus on purely political subcommunities, highlighted in the bottom panel of Fig. 1. Starting from the center-left wing, we can find a darker red community, including the main politicians of the Italian Democratic Party (*Partito Democratico*, or PD), its representatives in the European Parliament and some EU commissioners. The magenta group is instead mostly composed by the representatives of Italia Viva, a new party founded by the former Italian Prime Minister Matteo Renzi (December 2014–February 2016).

In turn, also the dark orange (M5S) community shows the presence of a purely political subcommunity (in orange in the bottom panel of Fig. 1), which contains the accounts of M5S politicians, parliament representatives and ministers. Also, we can find some journalists of *Il Fatto Quotidiano*, a newspaper supporting M5S.

Concerning the steel blue community, the purely political subcommunity of center-right and right wing parties (as Forza Italia, Lega and Fratelli d’Italia, from now on FI-L-FdI) is represented in blue in the bottom panel of Fig. 1.

Finally, the sky blue community is mainly composed by Italian embassies around the world.

We would to remark that, in Ref. [11], the authors exploited similar techniques to analyse the Italian debate on Twitter about migration policies. As in the current paper, after cleaning the system from random noise, the authors highlighted a group of coordinated accounts – a *bot squad* – increasing the visibility of a group of human-operated accounts. The division in community resembles the one found here, with few differences. First, in [11], media and center-right/right wing parties appeared in different communities from the very beginning; this is probably due to the fact that, in the present case, the criticism regarding the management of the pandemic by the main leaders of these parties were promptly reported by media. Secondly, in [11], M5S was not distinguishable from the right/center-right wing discursive community. This is not so surprising, since, at time of data collection of the previous manuscript, M5S was allied with Lega, the main right wing party in Italy. The data showed that M5S and Lega shared the same views on migration policies. In the present work, however, because Lega was no longer governing the country at time of data collection, and probably because of the difference in topics covered (immigration policies *versus* epidemic), M5S manifests its individuality.

### Analysis of domains – verified users

Here, we report a series of analyses related to the domains that mostly appear in the tweets of the validated network of verified users. We clarify that a domain, for us, corresponds to the so-called ‘second-level domain’ name,^5^ i.e., the name directly to the left of .com, .net, and any other top-level domains. For instance, repubblica.it, corriere.it, nytimes.com are considered as domains in the present manuscript. The domains have been tagged according to their degree of credibility and transparency, as indicated by the independent software toolkit NewsGuard https://www.newsguardtech.com/. The details of this procedure are reported below.

As a first step, we considered the network of verified accounts, whose communities and subcommunities have been shown in Fig. 1. On this topology, we labelled all domains that had been shared at least 20 times in tweets and retweets.

Table 2 shows the tags associated to the domains. In the rest of the paper, we shall be interested in quantifying reliability of news sources publishing during the period of interest. Thus, we will not consider those sources corresponding to social networks, marketplaces, search engines, institutional sites, etc.; nevertheless, the information regarding their frequency are available for the interested readers in the Additional file 1. Tags R, ∼R and NR in Table 2 are used only for news sites, be them newspapers, magazines, TV or radio social channels, and they stand for Reputable, Quasi Reputable, and Not Reputable, respectively.

**Table 2:** Tags used for domain labeling

Label	Description
R	Reputable news source
∼R	Quasi Reputable news source
NR	Not Reputable news source
S	social network
F	fundraiser and petition site
M	marketplace
P	official journal of a political party
IS	institutional site
ST	online streaming platform
SE	search engine
UNC	unclassified

As mentioned above, we relied on NewsGuard, a browser extension and mobile app resulting from the joint effort of journalists and software developers, aiming at evaluating news sites according to nine criteria concerning credibility and transparency. For evaluating the credibility level, the metrics consider, e.g., whether the news source regularly publishes false news, does not distinguish between facts and opinions, does not correct a wrongly reported news. For transparency, instead, the toolkit takes into account, e.g., whether owners, founders or authors of the news source are publicly known, and whether advertisements are easily recognizable. After combining the individual scores obtained out of the nine criteria, NewsGuard associates to a news source a global score from 1 to 100, where 60 is the minimum score for the source to be considered reliable. When reporting the results, the toolkit provides details about the criteria which passed the test and those that did not. For the sake of completeness, the Additional file 1 reports the procedure adopted by Newsguard journalists and editors to score each news site, the meaning of the score, and which are the textual information associated with the score, https://www.newsguardtech.com/ratings/rating-process-criteria/.

In order to have a sort of no-man’s land and not to be too abrupt in the transition between reputability and non-reputability, when the score was between 55 and 65, we considered the source to be quasi reputable, ∼R.

It is worth noting that not all the domains in the dataset under investigation were evaluated by NewsGuard at the time of our analysis. For those not yet evaluated by Newsguard, the annotation was made by three members of our team, who assessed the domains by using a subset of the NewsGuard criteria. The final class has been decided by majority voting (it never happened that the three annotators gave 3 different labels to the same domain). In the case of the network of verified users, considering only domains that appear at least 20 times, we have 80 domains annotated by Newsguard and 42 domains annotated by our three annotators. We computed the Fleiss’ kappa (*κ*) inter-rater agreement metric [44]. The metric measures the level of agreement of different annotators on a task. The annotators showed a moderate agreement for the classification of domains, with $\kappa = 0.63$.

Table 3 gives statistics about number and kind of tweets, the number of url and distinct url (dist url), the number of domains and users in the validated network of verified users. A url maintains here its standard definition^6^ and an example is http://www.example.com/index.html.

**Table 3:** Posts, urls, domains and users statistics in the validated network of verified users. “Tw” represent pure tweets, while “rt” indicates retweets. The number of tweets sharing an url is much higher than the one of retweets and it is a known results for verified users, from which they appear to drive the online debate

type	#post	#url	#dist url	#domain	#user
tw	46,277	37,095	32,605	1168	1115
rt	17,190	9796	7504	1178	1385

**Figure 2:**
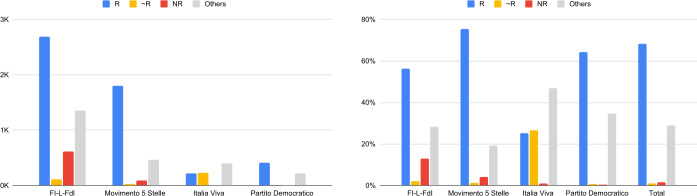
Number (left panel) and percentage (right panel) of Reputable, Circa Reputable, and Non Reputable news sites shared by the political subcommunities – Validated network of verified users

Figure 2 shows, on the left panel, the absolute value of Reputable, Quasi Reputable, Non Reputable shared domains, per political subcommunity. On the right panel, we can see a similar plot, but the results are given in terms of percentages. With ‘Others’, we denote all domains that do not refer to news sites, e.g., social networking sites, marketplaces, crowd sourcing platforms, etc. As can be seen from Table 2, Others include also the UNC class, i.e., that of domains appearing less than 20 times in the posts of the validated network of verified users. Indeed, there are many domains that occur only few times; for example, there are 300 domains that appear in the posts only once.

At a first glance, the majority of the news domains belong to the Reputable category.

Broadly speaking, we now examine the contribution of the different political parties, as represented on Twitter, to the spread of d/misinformation and propaganda.

Figure 2 clearly shows how the vast majority of the news coming from sources considered few or non reputable are shared by the center-right/right wing subcommunity (*FI-L-FdI*). Notably, the percentage of non reputable sources shared by the FI-L-FdI accounts is more than 30 times the second community in the NR ratio ranking. The impact of NR sources is even greater in absolute numbers, due to a major sharing activity of the users in this group (more than twice the value of the M5S subcommunity). Table 4 of the Additional file 1 gives more details on the annotation results.

Looking at Table 4, some peculiar behaviours can still be observed. Again, the center-right/right wing parties, while being the least represented ones in terms of users, are much more active than the other groups: each (verified) user is responsible, on average, of almost 77.86 messages, while the average is 23.96, 22.12 and 15.29 for M5S, IV and PD, respectively. It is worth noticing that IV, while being a recently founded party, is very active. Finally, the Additional file 1 reports an analysis of the hashtags used by the political subcommunities, in order to study the focus of the narratives within the various political groups.

**Table 4:** Posts, urls, domains and users statistics per political subcommunities – validated network of verified users: #post is the number of posts (divided in tweets and retweets), #url is the number of shared links, #dist url is the number of distinct urls, #domain is the number of distinct domains contained in all urls. While the number of (validated) verified users in the center-right/right wing subcommunity is lower than any other political group, their activity in writing original posts is at least twice greater than any other group. This difference is not present in the number of retweets

Subcommunity	#post	#url	#dist url	#domain	#user
only tweets					
FI-L-FdI	5031	4177	3728	210	62
Movimento 5 Stelle	2406	1839	1742	139	103
Italia Viva	943	458	417	96	69
Partito Democratico	736	370	353	74	60
only retweets					
FI-L-FdI	1587	582	510	151	72
Movimento 5 Stelle	997	546	469	104	103
Italia Viva	1048	399	348	147	82
Partito Democratico	747	273	258	94	88

### The validated retweet network

Here, we examine the *effective* retweet network, composed by users that retweet as a reaction to an interesting original tweet. As for effectiveness, we mean to consider the non random flow of messages from user to user. Indeed, it may happen that one tweet is shared either because it is viral, or because the retweeter is particularly active. Also, it could be that the account publishing the original tweet is extremely prolific. Instead, we are interested in the flow that cannot be explained only by the activity of users or by the popularity of the specific posts. Otherwise stated, our aim is to highlight the non-trivial sharing activity, distinguishing the relevant information from the random noise. We thus define a *directed* bipartite network in which one layer is composed by accounts and the other one by tweets. An arrow connecting a user *u* to a tweet *t* represents *u* writing the message *t*. An arrow in the opposite direction means that *u* is retweeting *t*. To filter out the random noise from this network, we make use of the directed version of the BiCM, i.e., the Bipartite Directed Configuration Model (*BiDCM* [46]), described in Sect. 6.2. BiDCM constrains the in- and out-degree sequences of nodes on both layers. In our scenario, these represent the users’ tweeting and retweeting activity and the virality of posts. In order to detect the non trivial flow of messages from user to user, for every (directed) couple of accounts, we compare the number of retweets observed in the real system with the expectation of the null-model. If the amount of retweets cannot be explained by the theoretical model, we project a link from the author to the retweeter in the monopartite directed network of users. Due to the process of validation, we call this network *directed validated projection*. More details can be found in Sect. 6.3.

The affiliation of unverified users to the various discursive communities is inferred exploiting the labels associated to verified users (see Sect. 4.1). The labels are propagated on the validated retweet network using the algorithm proposed in [47]. In Sect. 6 of the Additional file 1 we show that propagating labels on the entire weighted retweet network, on its binary version or on the validated version is almost equivalent in order to get the labels for the users in the directed validated network.

**Figure 3:**
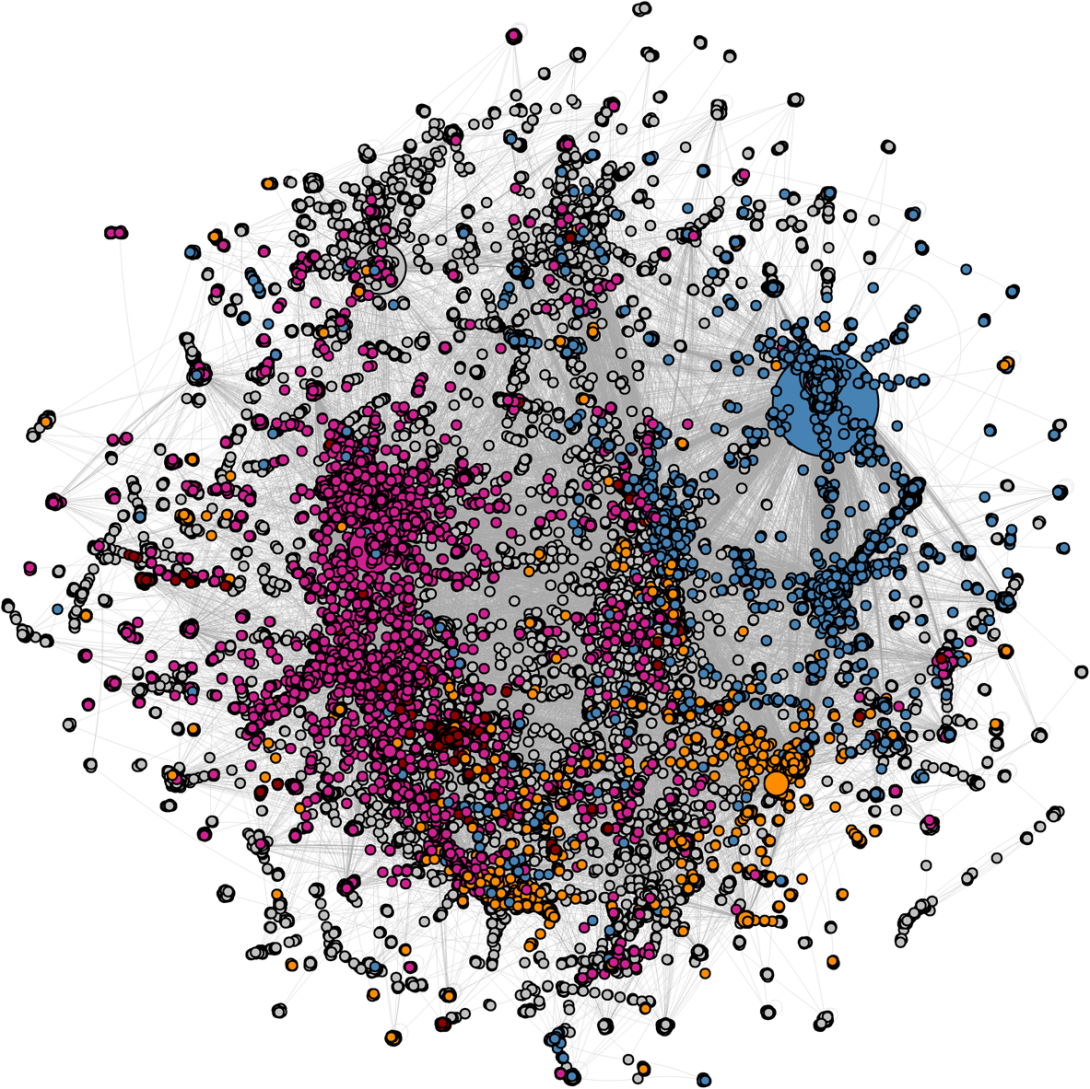
The directed validated projection of the retweet activity network: the communities have been highlighted according to the political discursive groups they take part to. All nodes not belonging to political discursive communities are in grey. Nodes’ dimensions are proportional to their out degree. The layout used for network visualization is the Distributed Recursive (Graph) Layout [45]

After applying the label propagation, we obtain the political communities in the validated retweet network, as shown in Fig. 3. We can see that the whole scenario changes dramatically with respect to the one of verified users. The center-right/right wing community is the most represented community in the whole network, with 11,063 users (representing 21.1% of all the users in the validated network), followed by Italia Viva users with 8035 accounts (15.4% of all the accounts in the validated network). The impact of M5S and PD is much more limited, with, respectively, 3286 and 564 accounts. It is worth noting that this result is unexpected, due to the recent formation of Italia Viva.

As in our previous study targeting the migration debate [11], the most effective users in terms of hub score [48] are almost exclusively from the center-right/right wing parties. Considering, e.g., the first 100 hubs, only 4 are not from these groups. Interestingly, 3 out of these 4 are verified users: Roberto Burioni, a popular Italian virologist, ranking 32nd; Agenzia Ansa, an Italian news agency, ranking 61st; and Tgcom24, the newscast of a private TV channel, ranking 73rd. The fourth account is an online news website, ranking 88th: this is an unverified account which belongs to a non political community.

Further, 3 in the top 5 hubs were already found in [11]. In particular, a journalist of a neo-fascist online newspaper (unverified user), an extreme right activist (unverified user) and the leader of Fratelli d’Italia, Giorgia Meloni (verified user), who ranks 3rd in the hub score. Matteo Salvini (verified user), who was the first hub in [11], ranks 9th, surpassed by his party partner Claudio Borghi (verified user), ranking 6th. The first hub in the present network is an (unverified) extreme right activist, posting videos against African migrants and accusing them to be responsible of the contagion and of violating lockdown measures.

#### Domain analysis on the directed validated network

**Figure 4:**
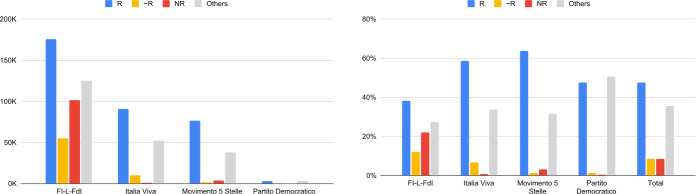
Number (left panel) and percentage (right panel) of Reputable, Circa Reputable, and Non Reputable news sites shared by the political subcommunities – Directed validated network

Figure 4 shows the annotation results of all the domains tweeted and retweeted by users in the directed validated network. The annotation was made considering the domains occurring at least 100 times. Even in this case, for those sites not yet evaluated by Newsguard, these have been annotated by the same three members of our team. We have 100 domains annotated by Newsguard and 53 domains annotated by the three annotators. Also in this case, the annotators showed a moderate agreement for the classification of domains, with $\kappa = 0.57$.

With respect to the annotation results for the network of verified users, the majority of URLs referring to news sources is still considered reputable, but its incidence is much reduced. Interestingly enough, the impact of at least nearly reputable sources is almost 19% for tweets and 16% for retweets, against percentages around 3% and 2% for the network of verified users.

The incidence of non reputable source in the subcommunity of center-right/right wing parties reaches the impressive percentage of 22.1%, which is even greater than what observed in Fig. 2 (i.e., 12.8%). The contribution of unverified users seems to boost the diffusion of unreliable content. It is even more alarming that the percentage of nearly reputable source is great too: considering both non reputable and nearly reputable sources the percentage is 34.2%. Thus, more than one third of the URLs shared in the validated network by FI-L-FdI is at least nearly reputable.

In absolute numbers, FI-L-FdI shares the highest number of NR URLs, being responsible of the 96% of NR URLs shared by all the political subcommunities. This behaviour is not only due to the greater amount of users: in the FI-L-FdI subcommunity, the accounts sharing NR URLs are particularly active. In this group, the average number of (original) NR posts sent per user is 32.21, which is almost 6 times the average for the M5S users (which has 5.38 NR posts per users); IV and PD have 4.48 and 1.00 as average, respectively. The frequency of accounts retweeting NR sources among all users from the same community is extremely high also for FI-L-FdI (57.6% for FI-L-FdI, 23.5% for M5S, 5.79% for IV and 2.5% for PD).

Table 5 reports statistics about posts, urls, distinct urls, users and verified users in the political subcommunities in the directed validated network. Noticeably, by comparing these numbers with those of Table 4, reporting analogous statistics about the validated network of verified users, we can see that now the number of retweets is much higher than that of tweets, and the opposite holds for verified user. Verified users tend to tweet more than retweet, while users in the directed validated network, which comprehends also unverified users, have a greater number of retweets, being even more than ∼5 times the one of tweets (depending on the community). This behaviour was already observed in [10, 11] and it is essentially due to the preeminence of verified users in shaping the public debate on Twitter. It is also remarkable the fact that verified users represent a minority of all users in the directed validated network.

**Table 5:** Posts, urls, domains and users statistics per political subcommunities – directed validated network. Differently from the case of verified users, the number of tweets is nearly one fifth of the number of retweets

Community	#post	#url	#dist url	#domain	#user	#verif
only tweets						
FI-L-FdI	176,137	95,902	63,710	3272	6831	56
Italia Viva	82,356	33,648	25,364	2243	4976	56
Movimento 5 Stelle	41,838	22,940	17,747	1536	1974	92
Partito Democratico	3247	1759	1671	277	337	51
only retweets						
FI-L-FdI	959,748	361,844	54,768	4304	10,749	48
Italia Viva	379,096	121,477	37,084	3915	7827	52
Movimento 5 Stelle	208,195	97,304	27,692	2647	3135	72
Partito Democratico	11,517	4424	3079	683	528	44

**Figure 5:**
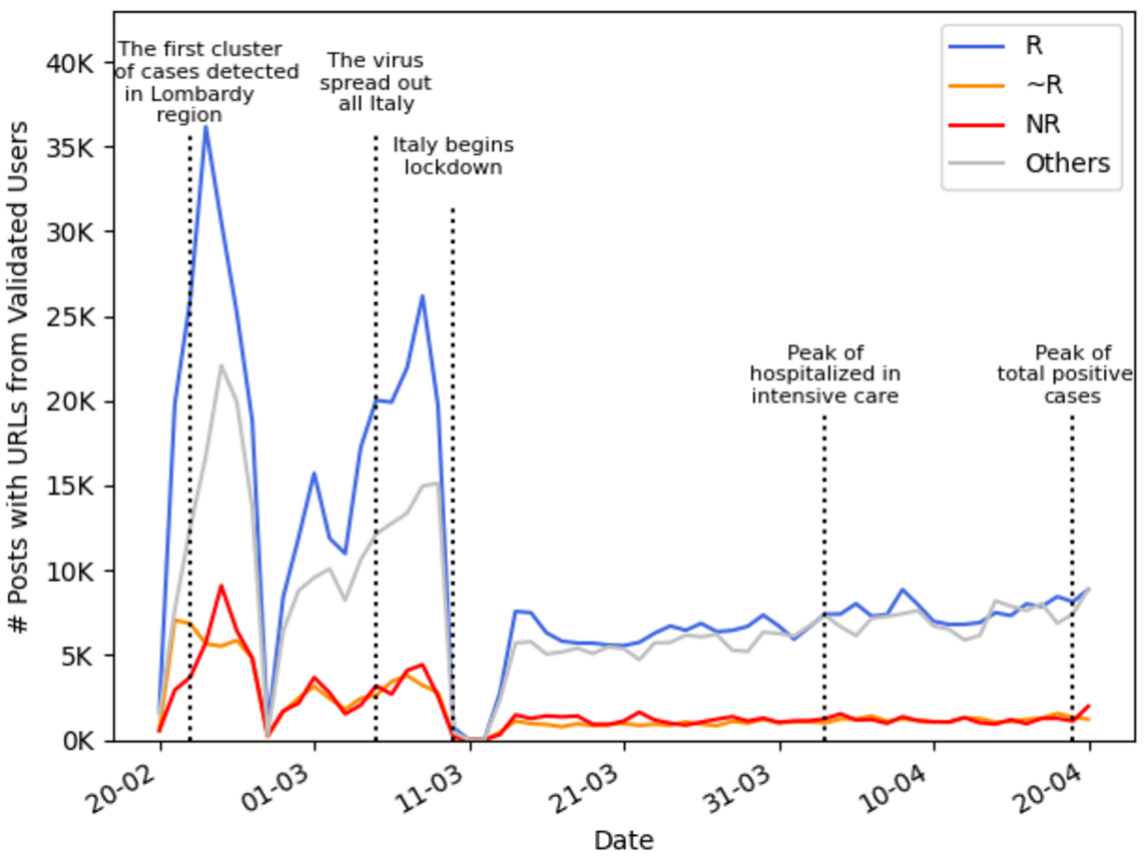
Domains’ spreading over time – validated directed network. The various main event regarding the pandemic have been reported in the plot. It is interesting to notice that the incidence of NR sources in the entire period is more or less constant in time. Interestingly enough, the same reduction of the overall activity after the beginning of the lockdown was detected even in [14, 22]

Figure 5 shows the trend of the number of posts containing URLs over the period of data collection. The highest peak appears after the discovery of the first cases in Lombardy. This corresponds to more than 68,000 posts containing URLs, but a higher traffic is still present before the beginning of the Italian lockdown, while there is a settling down as the quarantine went on.^7^ Interestingly, similar trends are present even in the analysis [14, 22].

It is interesting to note that the incidence of NR sources is nearly constant in the entire period.

### Non reputable domains shared in the effective flow of misinformation

As a final task, over the whole set of tweets produced or shared by the users in the directed validated network, we counted the number of times a message containing a URL was shared by users belonging to different political subcommunities, although without considering the semantics of the tweets. Namely, we ignored whether the URLs were shared to support or to oppose the presented arguments.

Table 6 shows the most frequent (tweeted and retweeted) NR domains shared by the political subcommunities; the number of occurrences is reported next to each domain.

**Table 6:** List of the most frequent NR domains, with relative occurrences, per political subcommunities. The count was made considering all posts for users of the direct validated network

FI-L-FdI		Italia Viva		Movimento 5 Stelle		Partito Democratico	
imolaoggi.it	16,041	dagospia.com	315	lantidiplomatico.it	1114	it.sputniknews.com	2
ilprimatonazionale.it	15,383	m.dagospia.com	134	m.dagospia.com	286	dagospia.com	2
voxnews.info	9334	imolaoggi.it	109	dagospia.com	266	laverita.info	1
stopcensura.info	8460	lantidiplomatico.it	72	it.sputniknews.com	98	lantidiplomatico.it	1
laverita.info	2647	ilprimatonazionale.it	61	imolaoggi.it	89	m.dagospia.com	1
stopcensura.org	2407	it.sputniknews.com	44	ilprimatonazionale.it	87	–	–
m.dagospia.com	2125	stopcensura.info	28	stopcensura.info	65	–	–
scenarieconomici.it	1647	agenpress.it	25	voxnews.info	46	–	–
it.sputniknews.com	1313	voxnews.info	25	agenpress.it	37	–	–
dagospia.com	1291	laverita.info	19	stopcensura.org	21	–	–
lantidiplomatico.it	1245	scenarieconomici.it	13	laverita.info	10	–	–
agenpress.it	1121	stopcensura.org	8	scenarieconomici.it	7	–	–
lavocedelpatriota.it	986	lavocedelpatriota.it	6	lavocedelpatriota.it	2	–	–

The first NR domains for FI-L-FdI refer to right, extreme right and neo-fascist propaganda. It is the case of imolaoggi.it, ilprimatonazionale.it and voxnews.info, recognised as disinformation websites by NewsGuard and by the two main Italian debunker websites, bufale.net and BUTAC.it.

As shown in the table, some domains, although in different number of occurrences, are present under more than one column, thus shared by users close to different political areas. However, since the semantics of the posts in which these domains are present were not investigated, the retweets of the links by more than one political subcommunity could be due to contrast, and not to support, the opinions present in the original posts. here, we intend to just present the most frequent NR domains.

## Discussion

Due to its impact on several dimensions of the society, the online debate regarding COVID-19 was the target of several early studies [12–15, 20–25]. In the present paper, we examine the presence of misinformation campaigns in the Italian online societal debate about the pandemic, during its peak of the first wave (end of February, 2020 – end of April, 2020). Our analysis is based on a general methodology reviewed in [17, 18] in order to extract both the discursive communities and the effective flow of messages [10, 11]: in particular, we build an entropy-based null-model, constraining part of the information of the real system, and we compare the observations on the real network with this benchmark.

The discursive communities are extracted focusing on verified users, i.e., public figures whose identity has been checked directly by Twitter. As in other studies [10, 11, 16], we observe that verified accounts lead the debate: their tweets are much more than their retweets. Due to such role, we examine in details the activity of verified users. Furthermore, we focus on the *effective* flow of information in the online debate: by comparing the system with an entropy-based null-model, we filter out all the random noise associated to the activity of users and virality of tweets. We highlight all the non trivial retweeting activities and further examine the properties of the filtered network, focusing on the incidence of non reputable news sources shared in the debate.

Despite the fact that the results have been achieved for a specific country, we believe that our approach, being general and unbiased by construction, is extremely useful to highlight non trivial properties and peculiarities. In particular, when analyzing the outcome of our investigation, some features attracted our attention:

*Persistence of clusters w.r.t. different discussion topics:* In Caldarelli et al. [11], we focused on tweets concerned with immigration, an issue that has been central in the Italian political debate for years. In particular, using the same techniques here adopted to extract the effective retweet network, we highlighted the presence of coordinated automated accounts increasing *effectively* the visibility of users belonging to the same discursive community. In this paper, we discover that the clusters and the echo chambers that were detected when analysing tweets about immigration are almost the same as those singled out when considering discussions about COVID-19.^8^ This may seem surprising, because a discussion about the pandemic may not be exclusively political, but also medical, economic and social. We can thus argue that the clusters are political in nature and, even when the topics change, users remain in their cluster on Twitter. (It is, in fact, well known that journalists and politicians use Twitter for spreading information and political propaganda, respectively).
  The reasons why political polarisation affect so strongly the vision of what should be an objective phenomenon is still an intriguing question.
*(Dis)Similarities amongst offline and online behaviors of members and voters of parties:* Maybe less surprisingly, the political habits is also reflected in the degree of participation to the online discussions. In particular, among the parties in the center-left wing side, a small party (Italia Viva) shows a much more effective social presence than the larger party of the Italian center-left wing (Partito Democratico), which has many more active members and more parliamentary representation. More generally, there is a significant difference in social presence among the different political parties, and the amount of activity is not at all proportional to the size of the parties in terms of members and voters.
*Spread of non reputable news sources:* In the online debate about the pandemic, many links to non reputable news sources are posted and shared. Kind and occurrences of the domains vary with respect to the corresponding political subcommunity. Furthermore, the center-right/right wing discursive community is characterised by a relatively small number of verified users that corresponds to a very large number of acolytes which are (on their turn) very active, three times as much as the ones of the opposite communities in the partition. In particular, when considering the amount of retweets from poorly reputable news sites, this community is by far (one order of magnitude) much more active than the others. As noted already in our previous publication [11], this extra activity could be explained by a more skilled use of the systems of propaganda – in that case a massive use of bot accounts and a targeted activity against migrants (as resulted from the analysis of the hub list).

While our work contributes to the literature regarding the analysis of the impact of misinformation on the online societal debate, it paves the way to other crucial analyses. In particular, it would be interesting to analyse the structure of the retweet network and how it may contribute to increase the visibility of some of the influential accounts that we detected (this was, in part, the target of the analysis in [49]). In this sense, even the role of automated accounts for the diffusion of NR news domains is of utmost importance in order to tackle the problem of online misinformation.

## Methods

In the present section we remind the main steps for the definition of an entropy-based null-model; the interested reader can refer to the review [18]. We start by revising the Bipartite Configuration Model [39], that has been used for detecting the network of similarities of verified users. We are then going to examine the extension of this model to bipartite *directed* networks [46]. Finally, we present the general methodology to project the information contained in a -directed or undirected- bipartite network, as developed in [40].

### Bipartite configuration model

Let us consider a bipartite network $\mathbf{G}^{*}_{\text{Bi}}$, in which the two layers are *L* and Γ. Define $\mathcal{G}_{\text{Bi}}$ the ensemble of all possible graphs with the same number of nodes per layer as in $\mathbf{G}^{*}_{\text{Bi}}$. It is possible to define the entropy related to the ensemble as [50]:

1 $$ S=-\sum_{\textbf{G}_{\text{Bi}}\in \mathcal{G}_{\text{Bi}}}P( \textbf{G}_{\text{Bi}}) \ln P(\textbf{G}_{\text{Bi}}), $$

where $P(\textbf{G}_{\text{Bi}})$ is the probability associated to the instance $\textbf{G}_{\text{Bi}}$. Now we want to obtain the maximum entropy configuration, constraining some relevant topological information regarding the system. For the bipartite representation of verified and unverified user, a crucial ingredient is the degree sequence, since it is a proxy of the number of interactions (i.e. tweets and retweets) with the other class of accounts. Thus in the present manuscript we focus on the degree sequence. Let us then maximise the entropy (1), constraining the average over the ensemble of the degree sequence. It can be shown, [40], that the probability distribution over the ensemble is

2 $$ P(\mathbf{G}_{\text{Bi}}) = \prod_{i,\alpha } (p_{i\alpha } )^{m_{i\alpha }} (1-p_{i\alpha } )^{1-m_{i\alpha }}, $$

where $m_{i\alpha }$ represent the entries of the biadjacency matrix describing the bipartite network under consideration and $p_{i\alpha }$ is the probability of observing a link between the nodes $i\in L$ and $\alpha \in \Gamma $. The probability $p_{i\alpha }$ can be expressed in terms of the Lagrangian multipliers *x* and *y* for nodes on *L* and Γ layers, respectively, as

3 $$ p_{i\alpha }=\frac{x_{i} y_{\alpha }}{1 + x_{i} y_{\alpha }}. $$

In order to obtain the values of *x* and *y* that maximize the likelihood to observe the real network, we need to impose the following conditions [51, 52]

4 $$ \textstyle\begin{cases} \langle k_{i} \rangle = \sum_{\alpha \in \Gamma } p_{i\alpha } = k_{i}^{*} & \forall i \in L \\ \langle k_{\alpha } \rangle = \sum_{i \in L} p_{i\alpha } = k_{\alpha }^{*} & \forall \alpha \in \Gamma . \end{cases}\displaystyle , $$

where the ∗ indicates quantities measured on the real network.

Actually, the real network is sparse: the bipartite network of verified and unverified users has a connectance $\rho \simeq 3.58\times 10^{-3}$. In this case the formula (3) can be safely approximated with the Chung–Lu configuration model, i.e.

$$ p_{i\alpha }\simeq x_{i}y_{\alpha }=\frac{k_{i}^{*}k_{\alpha }^{*}}{m}, $$

where *m* is the total number of links in the bipartite network.

### Bipartite directed configuration model

In the present subsection we will consider the case of the extension of the BiCM to *direct* bipartite networks and highlight the peculiarities of the network under analysis in this representation. The adjancency matrix describing a direct bipartite network of layers *L* and Γ has a peculiar block structure, once nodes are order by layer membership (here the nodes on *L* layer first):

5 $$ \textbf{A}= \left ( \textstyle\begin{array}{c|c} \textbf{O} & \textbf{M} \\ \hline \textbf{N}^{\text{T}}& \textbf{O} \end{array}\displaystyle \right ), $$

where the **O** blocks represent null matrices (indeed they describe links connecting nodes inside the same layer: by construction they are exactly zero) and **M** and **N** are non zero blocks, describing links connecting nodes on layer *L* with those on layer Γ and viceversa. In general $\textbf{M}\neq \textbf{N}$, otherwise the network is not distinguishable from an undirected one.

We can perform the same machinery of the section above, but for the extension of the degree sequence to a directed degree sequence, i.e. considering the in- and out-degrees for nodes on the layer *L*,

6 $$ k_{i}^{\text{out}}=\sum_{\alpha \in \Gamma }m_{i\alpha } \quad \text{and}\quad k_{i}^{\text{in}}=\sum _{\alpha \in \Gamma }n_{i\alpha } $$

(here $m_{i\alpha }$ and $n_{i\alpha }$ represent respectively the entry of matrices **M** and **N**) and for nodes on the layer Γ,

7 $$ k_{\alpha }^{\text{out}}=\sum_{i \in L}n_{i\alpha } \quad \text{and} \quad k_{\alpha }^{\text{in}}=\sum _{i \in L}m_{i\alpha }. $$

The definition of the Bipartite *Directed* Configuration Model (BiDCM, [46]), i.e. the extension of the BiCM above, follows closely the same steps described in the previous subsection. Interestingly enough, the probabilities relative to the presence of links from *L* to Γ are independent on the probabilities relative to the presence of links from Γ to *L*. If $q_{i\alpha }$ is the probability of observing a link from node *i* to node *α* and $q'_{i\alpha }$ the probability of observing a link in the opposite direction, we have

8 $$ q_{i\alpha }= \frac{x_{i}^{\text{out}}y_{\alpha }^{\text{in}}}{1+x_{i}^{\text{out}}y_{\alpha }^{\text{in}}} \quad \text{and}\quad q'_{i\alpha }= \frac{x_{i}^{\text{in}}y_{\alpha }^{\text{out}}}{1+x_{i}^{\text{in}}y_{\alpha }^{\text{out}}}, $$

where $x_{i}^{\text{out}}$ and $x_{i}^{\text{in}}$ are the Lagrangian multipliers relative to the node $i\in L$, respectively for the out- and the in-degrees, and $y_{\alpha }^{\text{out}}$ and $y_{\alpha }^{\text{in}}$ are the analogous for $\alpha \in \Gamma $.

In the present application we have some simplifications: the bipartite directed network representation describes users (on one layer) writing and retweeting posts (on the other layer). If users are on the layer *L* and posts on the opposite one and $m_{i\alpha }$ represents the user *i* writing the post *α*, then $k_{\alpha }^{\text{in}}=1\ \forall \alpha \in \Gamma $, since each message cannot have more than an author. Notice that, since our constraints are conserved on average, we are considering, in the ensemble of all possible realisations, even instances in which $k_{\alpha }^{\text{in}}>1$ or $k_{\alpha }^{\text{in}}=0$, or, otherwise stated, non physical; nevertheless the average is constrained to the right value, i.e. 1. The fact that $k_{\alpha }^{\text{in}}$ is the same for every *α* allows for a great simplification of the probability per link on **M**:

9 $$ q_{i\alpha }=\frac{(k_{i}^{\text{out}})^{*}}{N_{\Gamma }}, $$

where $N_{\Gamma }$ is the total number of nodes on the Γ layer. The simplification in (9) is extremely helpful in the projected validation of the bipartite directed network [10].

### Validation of the projected network

**Figure 6:**
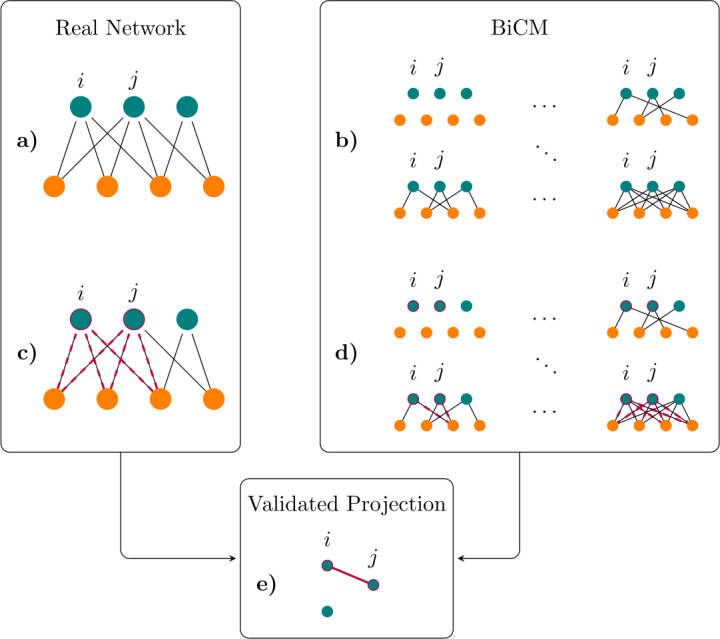
Schematic representation of the projection procedure for bipartite undirected networks. (**a**) An example of a real bipartite network. For the actual application, the two layers represent verified (turquoise) and unverified (gray) users and a link between nodes of different layers is present if one of the two users retweeted the other one, at least once. (**b**) Definition of the Bipartite Configuration Model (BiCM) ensemble. Such ensemble includes all possible link realisations, once the number of nodes per layers has been fixed. (**c**) we focus our attention on nodes *i* and *j*, i.e., two verified users, and count the number of common neighbours (in magenta both the nodes and the links to their common neighbours). Subsequently, (**d**) we compare this measure on the real network with the one on the ensemble: If this overlap is statistically significant with respect to the BiCM, (**e**) we have a link connecting the two verified users in the projected network. The figure is an adaptation from [11]

The information contained in a bipartite -directed or undirected- network, can be projected onto one of the two layers. The rationale is to obtain a monopartite network encoding the non trivial interactions among the two layers of the original bipartite network. The method is pretty general, once we have a null-model in which probabilities per link are independent, as it is the case of both BiCM and BiDCM [40]. The method is graphically depicted in Fig. 6 in the case of BiCM; the case of BiDCM is analogous.

The first step is represented by the definition of a bipartite motif that may capture the non trivial similarity (in the case of an undirected bipartite network) or flux of information (in the case of a directed bipartite network). This quantity can be captured by the number of *V*-motifs between users *i* and *j* [39, 53],

10 $$ V_{ij} = \sum_{\alpha \in \Gamma } m_{i\alpha }m_{j\alpha }, $$

or by its direct extension

11 $$ \mathcal{V}_{ij}=\sum_{\alpha \in \Gamma }m_{i\alpha }n_{\alpha j} $$

(note that $\mathcal{V}_{ij}\neq \mathcal{V}_{ji}$). We compare the abundance of these motifs with the null-models defined above: all motifs that cannot be explained by the null-model, i.e. whose p-value are statistically significance, are validated into the projection on one of the layers [40].

In order to assess the statistically significance of the observed motifs, we calculate the distribution associated to the various motifs. For instance, the expected value for the number of V-motifs connecting *i* and *j* in an undirected bipartite network is

12 $$ \langle V_{ij} \rangle = \sum_{\alpha \in \Gamma } p_{i \alpha } p_{j\alpha }, $$

where $p_{i\alpha }$s are the probability of the BiCM. Analogously,

13 $$ \langle \mathcal{V}_{ij} \rangle = \sum _{p \in P} q_{i \alpha } q'_{j\alpha }= \frac{(k_{i}^{\text{out}})^{*} (k_{j}^{\text{in}})^{*}}{N_{\Gamma }}, $$

where in the last step we use the simplification of (9) [10].

In both the direct and the undirect case, the distribution of the V-motifs or of the directed extensions is Poisson Binomial one, i.e. a binomial distribution in which each event shows a different probability. In the present case, due to the sparsity of the analysed networks, we can safely approximate the Poisson-Binomial distribution with a Poisson one [54].

In order to state the statistical significance of the observed value, we calculate the related p-values according to the relative null-models. Once we have a p-value for every detected V-motif, the related statistical significance can be established through the False Discovery Rate (*FDR*) procedure [55], which, respect to other multiple test hypothesis, controls the number of False Positives. In our case, all rejected hypotheses identify the amount of V-motifs that cannot be explained only by the ingredients of the null model and thus carry non trivial information regarding the systems. In this sense, the validated projected network includes a link for every rejected hypothesis, connecting the nodes involved in the related motifs.

## Supplementary Information

Below is the link to the electronic supplementary material.

Supplementary information (PDF 2.2 MB)

## Data Availability

Twitter data can be downloaded as a list of the tweet ids used in the analysis from the website of the TOFFEe project at https://toffee.imtlucca.it/datasets, in agreement with Twitter policy (https://developer.twitter.com/en/developer-terms/more-on-restricted-use-cases). NewsGuard data are proprietary and cannot be shared.

## Footnotes

1 In Italy, since the beginning of the pandemic and at time of writing, almost 4 million persons have contracted the virus: of these, more than 119k have died. Source: http://www.protezionecivile.gov.it/ Accessed April 26, 2021.

2 We had an interruption of one day and 4 hours on February 27th, 2020, and another of three days and 8 hours on March 10th, 2020, due to a connection breakdown. However, because of the validation procedure that we applied on the aggregated network over the entire period of data collection (see Sects. 4 and 6), we expect the effect of the breakdown to be negligible for the results evaluation.

3 Specifically, during peaks of traffic at the end of February, 2020, we ran into Twitter rate limits less than once a day.

4 The term *validated* should not be confused with the term *verified*, which instead denotes a Twitter user who has passed the formal Twitter verification procedure.

5 https://en.wikipedia.org/wiki/Domain_name

6 https://en.wikipedia.org/wiki/URL

7 The low peaks for February 27 and March 10 are due to an interruption in the data collection, caused by a connection breakdown.

8 Actually, in [11] the center-right/right wing parties were distinct from the Media community. Here, we found them distinct after launching, on the first community, a further community detection algorithm.
