# Supplementary material for: Flow of online misinformation during the peak of the COVID-19 pandemic in Italy
Source: EPJ Data Sci. 2021 Jul 6;10(1):34. doi: 10.1140/epjds/s13688-021-00289-4 (PMC8258478; doi:10.1140/epjds/s13688-021-00289-4)
Supplement: Supplementary file 1 — Supplementary information (PDF 2.2 MB) [file 13688_2021_289_MOESM1_ESM.pdf]

RESEARCH

Flow of online misinformation during the peak of the COVID-19 pandemic in Italy Supplementary Material

Guido Caldarelli[1,2,3*†], Rocco De Nicola[3†], Marinella Petrocchi[4†], Manuel Pratelli[3†] and Fabio Saracco[3†]

*Correspondence:
Guido.Caldarelli@unive.it
[1] Department of Molecular Sciences and Nanosystems, Ed. Alfa, Via Torino 155, 30170 Venezia Mestre, Italy
Full list of author information is available at the end of the article
†Equal contributor

1 Italian socio-political situation during the period of data collection

Here, we present the social context in which our analysis is set. This subsection is divided into two parts: the contagion evolution and the political situation. These two aspects are closely related.

1.1 Evolution of the Covid-19 pandemic in Italy

A first Covid-19 outbreak was detected in Codogno, Lodi, Lombardy region, on February 19th, 2020[1]. In the very next day, two cases were detected in Vò, Padua, Veneto region. On February 22nd, in order to contain the contagions, the government decided to put in quarantine 11 municipalities, 10 in the area around Lodi and Vò[2]. Nevertheless, the number of contagions raised to 79, hitting 5 different regions; one of the infected person in Vò died, representing the first registered Italian Covid-19 victim[3]. On February 23rd, there were already 229 confirmed cases in Italy. The first lockdown should have lasted until the 6th of March, but due to the still increasing number of contagions in northern Italy, the Italian Prime Minister Giuseppe Conte intended to extend the quarantine zone to almost all the northern Italy on Sunday, March 8th[4]: traveling to and from the quarantine zone was limited to case of extreme urgency. A draft of the decree announcing the expansion of the quarantine area appeared on the website of the Italian newspaper *Corriere della Sera* on the late evening of Saturday 7th, causing some panic in the interested areas[5]: around 1000 people, living in Milan, but coming from southern regions, took trains and planes to reach their place of

[1] Prima Lodi, "“Paziente 1”, il merito della diagnosi va diviso... per due", 8th June 2020
[2] Italian Gazzetta Ufficiale, "DECRETO-LEGGE 23 Febbraio 2020, n. 6". The date is intended to be the very first day of validity of the decree.
[3] Il Fatto Quotidiano, "Coronavirus, è morto il 78enne ricoverato nel Padovano. 15 contagiati in Lombardia, un altro in Veneto", 22nd February 2020.
[4] BBC News, "Coronavirus: Northern Italy quarantines 16 million people", 8th March 2020"
[5] The Guardian, "Leaked coronavirus plan to quarantine 16m sparks chaos in Italy", 8th March 2020

origin[6][7]. In any case, the new quarantine zone covered the entire Lombardy and partially other 4 regions.

Remarkably, close to Bergamo, Lombardy region, a new outbreak was discovered and the possibility of defining a new quarantine area on March 3rd was considered: this opportunity was later abandoned, due to the new northern Italy quarantine zone of the following days. This delay seems to have caused a strong increase in the number of contagions, making the Bergamo area the most affected one, in percentage, of the entire country[8]; at time of writing, there are investigations regarding the responsibility of this choice.
On March 9th, the lockdown was extended to the whole country, resulting in the first country in the world to decide for national quarantine[9]. Travels were restricted to emergency reasons or to work; all business activities that were not considered as essentials, as pharmacies and supermarkets, had to be closed. Until the 21st of March, lockdown measures became progressively stricter all over the country. Starting from the 14th of April, some retails activities, as children clothing shops, reopened. A first fall in the number of deaths was observed on the 20th of April[10]. A limited reopening started with the so-called "Fase 2" (*Phase 2*) on the 4th of May[11].
From the very first days of March, the limited capacity of the intensive care departments caused a re-organization of Italian hospitals, leading, e.g., to the opening of new intensive care departments[12]. Moreover, new communication forms with the patient relatives were proposed, new criteria for the intubated patients were developed, and, in the extreme crisis, in the most infected cases, the emergency management took to give priority to the hospitalisation to patients with a higher probability to recover[13].

Outbreaks were mainly present in hospitals [1]. Unfortunately, healthcare workers were contaminated by the virus[14]. This contagion resulted in a relative high number of fatalities: by the 22nd of April, 145 Covid deaths were registered among

---

[6] il Messaggero, "Coronavirus, a Milano la fuga dalla "zona rossa": folla alla stazione di Porta Garibaldi", 8th March 2020"
[7] repubblica.it, "Coronavirus, l'illusione della grande fuga da Milano. Ecco i veri numeri degli spostamenti verso sud", 23rd April 2020
[8] sky.com, "Coronavirus: Italian army called in as crematorium struggles to cope with deaths", 19th March 2020.
[9] BBC News, "Coronavirus: Italy extends emergency measures nationwide", 10th March 2020
[10] Al Jazeera, "Italy sees first fall of active coronavirus cases: Live updates", 20th April, 2020.
[11] Repubblica.it, "Coronavirus in Italia, verso primo ok spostamenti dal 4/5, non tra Regioni. Conte: "Non è un liberi tutti"", 22nd April 2020
[12] The New York Times, "Italy's Health Care System Groans Under Coronavirus — a Warning to the World", 12th March 2020.
[13] Il Corriere della Sera, "Coronavirus, il medico di Bergamo: "Negli ospedali siamo come in guerra. A tutti dico: state a casa"", 9th March 2020.
[14] Ansa.it, "Coronavirus: Ordini degli infermieri, 4 mila i contagiati", 29th March 2020.

doctors. Due to the pressure on the intensive care capacity, even the healthcare personnel was subject to extreme stress, especially in the most affected areas[15].

1.2 Italian political situation during the pandemic

On August 8th 2019, the leader of Lega, the main Italian right wing party, announced to negate the support to the government of Giuseppe Conte, which was formed after a post-election coalition between the Movement 5 Stars -M5S- and the Lega. The Prime Minister Giuseppe Conte resigned on the 20th of August and opened to the political crisis. After few days of negotiation, M5S, the most represented party in the Italian parliament, agreed to form a new government with the Italian Democratic Party (*Partito Democratico*, PD). PD, on the other hand, agreed, upon the suggestion of the former secretary and Prime Minister Matteo Renzi. After the formation of the new government, again led by Giuseppe Conte, Matteo Renzi formed a new center-left party, *Italia Viva* (*Italy alive*, IV), due to some discord with PD; despite the split, Italia Viva continued to support the actual government, having some of its representatives among the ministers and undersecretaries, but often marking its distance with respect to both Pd and M5S. Due to the great impact that Matteo Salvini and Giorgia Meloni -leader of Fratelli d'Italia, a right wing party- had on social media, they started a massive campaign against the government the day after its inauguration.
The regions of Lombardy, Veneto, Piedmont and Emilia-Romagna experienced the highest number of contagions during the pandemic; among those, the former 3 were administrated by the right and center-right wing parties, the fourth one by the PD. The disagreement in the management of the pandemic between regions and the central government was the occasion to exacerbate the political debate (in Italy, regions have a quite wide autonomy for healthcare). The regions administrated by the right wing parties criticised the centrality of the decisions regarding the lock down, while the national government criticised the health management (in Lombardy, the healthcare system has a peculiar organisation, in which the private sector is supported by public funding) and its ineffective measure to reduce the number of contagions. The debate was ridden even at a national level: the opposition criticized the financial origin of the support to the various economic sectors. Moreover, the role of the European Union in providing funding to recover Italian economics after the pandemic was debated.

2 Composition of the subcommunities in the validated network of verified Twitter users

Here, we detail the composition of the communities shown in Figure 1 of the main text. We remind the reader that, after applying the Louvain algorithm to the validated network of verified Twitter users, we could observe 4 main communities, that correspond to

1. Center right/Right wing parties and media (in steel blue);
2. Center-left wing (dark red);
3. 5 Stars Movement (*M5S*) (in dark orange);

[15] Internazionale, "Il dolore invisibile dei medici in corsia contro il coronavirus", 1st April 2020.

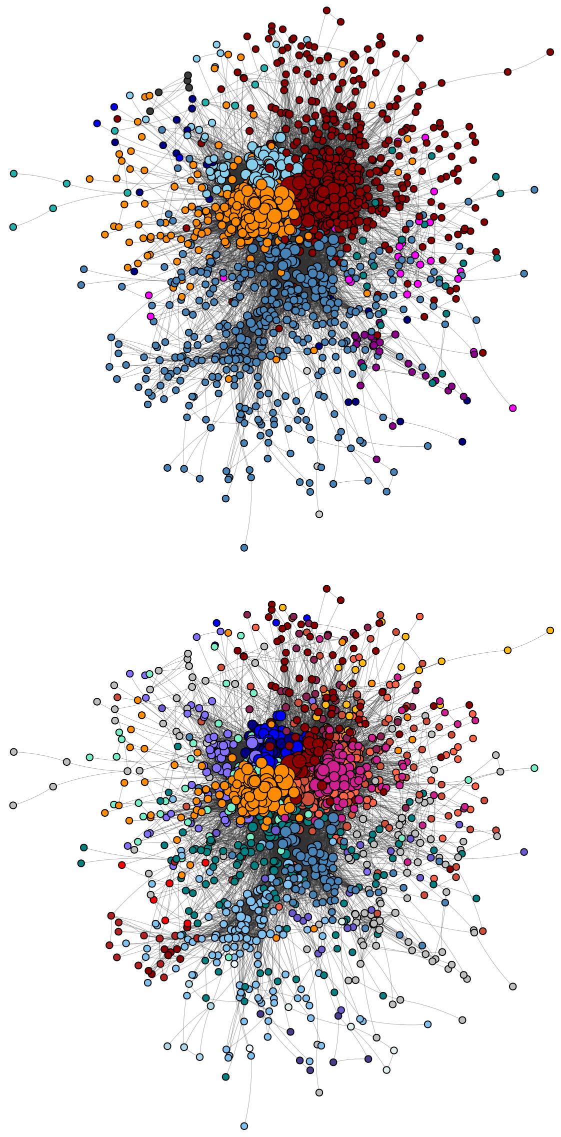

Figure 1 Validated projection of the bipartite network of verified/unverified accounts. In the top panel, the monopartite projection in which just communities are displayed. In the bottom panel, the subcommunities, obtained by rerunning the Louvain algorithm in each of the former 4 main communities.

4 Institutional accounts (in sky blue).

Starting from the center-left wing, we can find a slightly lighter red community, including various NGOs (the Italian chapter of UNICEF, Medecins Sans Frontieres, Action Aid, Emergency, Save the Children), various left-oriented journalists, VIPs and pundits[16]. Finally, we can find in this group political movements ('6000sardine') and politicians on the left of PD (as Giuseppe Civati,

[16]As the cartoonists Makkox and Vauro, the singers Marracash, FrankieHiNRG, Ligabue and "il Volo" vocal band, and journalists from Repubblica (Ezio Mauro, Carlo Verdelli, Massimo Giannini), from La7 TV channel (Riccardo Formigli, Diego Bianchi).

Pietro Grasso, Ignazio Marino) or on the left current of the PD (Laura Boldrini, Michele Emiliano, Stefano Bonaccini). A darker red sub-community turns out to be composed by the main politicians of the Italian Democratic Party (PD), as well as by representatives of the European Parliament (Italians and others) and some EU commissioners. The magenta group is mostly composed by the representatives of the newly founded Italia Viva, by the former Italian Prime Minister Matteo Renzi (December 2014 - February 2016) and former secretary of PD. In golden red, we can find the subcommunity of Catholic and Vatican groups. Finally, the dark violet red and light tomato subcommunities are composed mainly by journalists. Interestingly enough, the dark violet red contains also accounts related to the city of Milan (the major, the municipality, the public services account) and to the spoke person of the Chinese Minister of Foreign Affair.

In turn, also the orange (M5S) community shows a clear partition in substructures. In particular, the dark orange subcommunity contains the accounts of politicians, parliament representatives and ministers of M5S, as well as journalists close to the party, and the official account of *Il Fatto Quotidiano*, a newspaper explicitly supporting the M5S. Since one of the main leaders of the Movement, Luigi Di Maio, was also the Italian Minister of Foreign Affairs, we can find in this subcommunity also the accounts of several Italian embassies around the world, as well as the account of the Italian representatives at NATO, OCSE and OAS. In aquamarine, we can find the official accounts of some private and public, national and international, health institutes (as the Italian Istituto Superiore di Sanità, literally the *Italian National Health Institute*, the World Health Organization, and the Fondazione Veronesi), the Minister of Health Roberto Speranza, and some foreign embassies in Italy. Finally, in the Light Slate Blue subcommunity, we can find various Italian ministers as well as the Italian police and army forces.

Similar considerations apply to the steel blue community. In steel blue, the subcommunity of center-right/right wing parties (as Forza Italia, Lega and Fratelli d'Italia). The presidents of Lombardy, Veneto and Liguria, administrated by center-right/right wing parties, can be found here. (In the following this subcommunity is going to be called as FI-L-FdI, recalling the initials of the political parties contributing to this group.) The sky blue subcommunity includes the national federations of various sports, the official accounts of athletes and sport players (mostly soccer players) and their teams, as well as sport journals, newscasts and journalists. The teal subcommunity contains the main Italian news agencies, some of the main national and local newspapers, newscasts and their journalists. In this subcommunity, there are also accounts of many universities; finally, it includes also local public service newscasts. The firebrick subcommunity contains accounts related to the AS Roma football club; analogously, in dark red, official accounts of AC Milan and its players. The slate blue subcommunity is mainly composed by the official accounts of radio and TV programs of Mediaset, the main private Italian broadcasting company, together with singers and musicians. Other smaller subcommunities include other sport federations and sports pundits.

Finally, the sky blue community is mainly composed by Italian embassies around the world. The navy subpartition contains also the official accounts of the

President of the Republic, the Italian Minister of Defense and the one of the Commissioner for Economy at EU and former Prime Minister, Paolo Gentiloni.

3 Domain analysis for the validated network of verified users

label	description
R	Reputable news source
∼ R	Quasi Reputable news source
NR	Not Reputable news source
S	social network
F	fundraiser and petition site
M	marketplace
P	official journal of a political party
IS	institutional site
ST	online streaming platform
SE	search engine
UNC	unclassified

Table 1 Tags used for labeling the domains. Label UNC is assigned to those domains with less than 20 occurrences in the dataset. This figure is inherited from the main text.

Table 2 shows the percentage of the different types of domains for the 4 communities identified in the top panel of Fig. 1.

Community	#url	R	∼R	NR	S	F	M	P	IS	ST	SE	UNC
`only tweets`												
steel blue	22029	74.5	0.9	2.7	3.3	0.1	0.0	0.7	0.0	0.0	0.0	17.8
dark red	9185	79.0	2.0	0.1	1.6	0.1	0.0	0.6	0.3	0.0	0.0	16.3
dark orange	3437	54.1	0.2	0.2	6.1	0.1	0.0	0.9	1.6	0.3	0.0	36.5
sky blue	1106	65.8	0.0	0.0	6.2	0.0	0.0	0.1	0.0	0.0	0.0	27.9
`only retweets`												
steel blue	2481	69.7	0.9	3.2	4.4	0.4	0.0	0.1	0.0	0.0	0.0	21.3
dark red	3563	71.4	1.9	0.1	3.7	0.4	0.0	0.6	0.6	0.0	0.0	21.3
dark orange	2202	41.0	0.5	0.9	8.7	0.4	0.0	0.6	1.4	0.7	0.0	45.8
sky blue	1051	38.3	1.5	0.1	12.7	0.3	0.0	0.1	0.8	0.0	0.1	46.1

Table 2 Annotation per communities – validated network of verified users. The colors are those of the greatest communities of the top panel of Fig. 1. Steel blue represent the discursive community of Media and center-right/right wing parties; in dark red, the center-left wing parties and their supporters; in dark orange, the supporters of Movimento 5 Stelle and, in sky blue, the official government accounts. The presence of many more tweets than retweets may be surprising: actually, it is typical of verified users focusing their production in original messages, as already observed in [2–4].

Table 3 shows that the steel blue community (including both politicians and Media) is the most active one, even if it is not the most represented: the number of users is lower than the one of the center-left community (the biggest one, in terms of numbers), but the number of posts containing a valid url is almost the double of that of the center-left group. The activity of steel blue verified users is more focused on content production (see the `only tweets` sub-table) than on sharing (see the `only retweets` sub-table). Retweets represent almost 14.6% of all posts from Media and right wing community, while in the case of the center-left community the value is 34.5%. This effect is observable even in the average `only tweets` post per verified user: a right-wing user and a Media user have an average of 88.75 original posts, against 34.27 for center-left users. These numbers are probably due to the presence, in the former community, of the Italian most accessed media, that spread their (original) pieces of news on Twitter.
Table 4 shows the domain annotation per political sub-communities. The presence of urls from a non reputable source in the steel blue community is more than 10

Community	#post	#url	#dist url	#domain	#user
steel blue	30877	24510	20718	648	417
dark red	17202	12748	10999	744	452
dark orange	8990	5639	4389	640	316
sky blue	3897	2157	1626	348	149
`only tweets`					
steel blue	26359	22029	19222	467	297
dark red	11275	9185	8435	430	329
dark orange	5240	3437	3042	351	245
sky blue	1738	1106	964	143	114
`only retweets`					
steel blue	4518	2481	2175	348	328
dark red	5927	3563	3050	483	399
dark orange	3750	2202	1633	423	264
sky blue	2159	1051	740	269	147

Table 3 Posts, urls, domains and users statistics per communities – validated network of verified users. The frequency of posts in the steel blue community is originated by the presence of Media in this group. Nevertheless, as we will see in Table 4, even the political subcommunity contained in the steel blue group is particular prolific.

times higher than the second score in the same field for (`only tweets`). It is worth noting that, for the case of the dark orange and sky blue communities, which are smaller both in terms of users and number of posts, the presence of non classified sources is quite strong (it represents nearly 46% of the posts retweeted, for both the communities), as it is the frequency of posts linking to social network contents. Interestingly enough, verified users of both groups seem to focus slightly more on the same domains: there are, on average, 1.59 and 1.80 posts for each url domain, respectively for the dark orange and sky blue communities, and, on average, 1.26 and 1.34 posts for the steel blue and the dark red communities.

Subcommunity	#url	R	∼R	NR	S	F	M	P	IS	ST	SE	UNC
FI-L-FdI	4759	56.4	2.3	12.8	14.5	0.1	0.0	3.1	0.0	0.0	0.0	10.8
Movimento 5 Stelle	2385	75.5	0.1	0.4	6.6	0.0	0.0	1.9	0.0	1.1	0.0	14.4
Italia Viva	857	25.3	26.6	0.1	10.0	0.7	0.1	8.4	0.5	0.0	0.0	28.3
Partito Democratico	643	64.4	0.6	0.3	9.2	0.8	0.0	0.0	3.6	0.0	0.2	20.9
`only tweets`												
FI-L-FdI	4177	59.0	2.1	13.0	14.6	0.0	0.0	3.5	0.0	0.0	0.0	7.8
Movimento 5 Stelle	1839	79.4	0.1	0.4	6.3	0.0	0.0	1.7	0.0	0.6	0.0	11.5
Italia Viva	458	19.2	39.1	0.2	9.0	0.2	0.2	11.4	0.0	0.0	0.0	20.7
Partito Democratico	370	71.9	0.5	0.5	5.4	1.1	0.0	0.0	3.8	0.0	0.0	16.8
`only retweets`												
FI-L-FdI	582	38.0	3.4	11.7	14.1	0.3	0.0	0.3	0.0	0.0	0.0	32.2
Movimento 5 Stelle	546	62.3	0.2	0.4	7.9	0.2	0.0	2.6	0.0	2.9	0.0	23.5
Italia Viva	399	32.3	12.3	0.0	11.3	1.3	0.0	5.0	1.0	0.0	0.0	36.8
Partito Democratico	273	54.2	0.7	0.0	14.3	0.4	0.0	0.0	3.3	0.0	0.4	26.7

Table 4 Domains annotation per political subcommunities - validated network of verified users. The incidence of reputable sources strongly reduces in the retweets for all the subcommunities, but Italia Viva. We argue that verified users are more cautious when writing their original messages, while they are more relaxed when sharing other messages. The references to Social Networks (S) are relatively strong in all the subcommunities.

3.1 Hashtags by verified users

Figures 2 and 3 report statistics about the most diffused hashtags in the 4 political subcommunities. Actually, from the various hashtags, we can derive important information regarding the political discursive communities and their view about the pandemic and its management. First, M5S is the greatest user of hashtags: the two most used hashtags have been used almost twice the most used

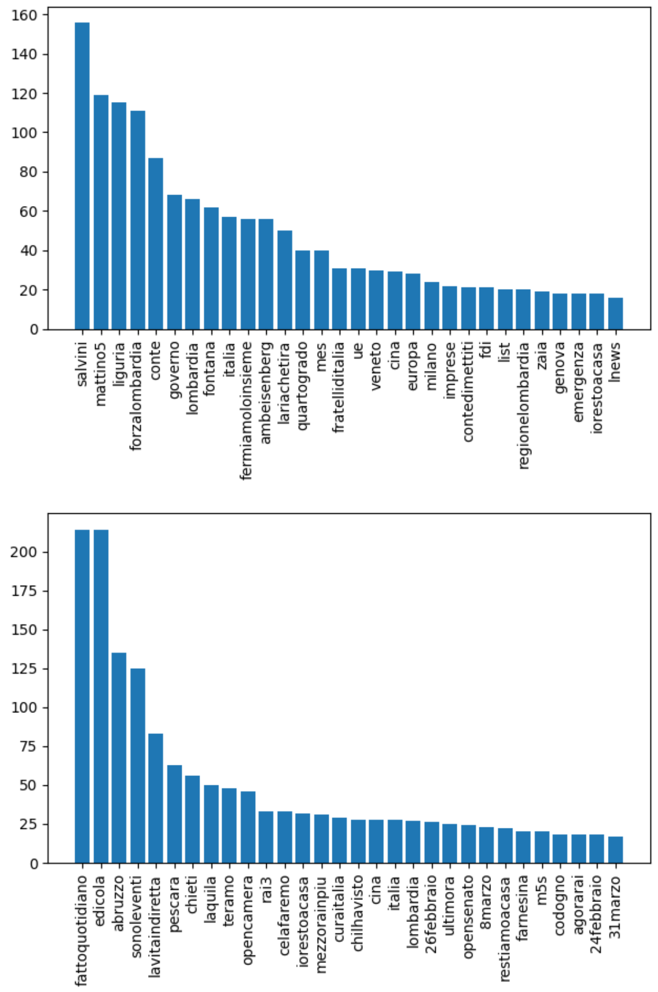

Figure 2 The 30 most diffused hashtags in the political sub-communities. Verified users. Top panel: right and center-right wing; bottom panel: 5 Stars Movement.

hashtags by PD, for instance. This heavy usage is probably due to the presence in this community of journalists and of the official account of *Il Fatto Quotidiano*, a newspaper explicitly supporting M5S: indeed, the first two hashtags are “#ilfattoquotidiano” and “#edicola” (*kiosk*, in Italian).

There is a relevance of hashtags intended to encourage the population during the lockdown: it is the case of “#celafaremo” (*we will make it*), “#iorestoacasa” (*I am staying home*), “#fermiamoloinsieme” (*Let’s stop it together*): “#iorestoacasa” is present in every community, but it ranks 13th in the M5S political community, 29th in the FI-L-FdI community, 2nd in the Italia Viva community and 10th in the PD one. Remarkably, “#celafaremo” is present only in the M5S group, as “#fermiamoloinsieme” can be found in the top 30 hashtags only in the center-right/right wing cluster. The PD, being present in various European institutions, mentions more European Union related hashtags (“#europeicontrocovid19”, *Europeans against covid-19*), in order to ask for a common reaction of the EU. The center-right/right wing community has other

hashtags as “#forzalombardia” (*Go, Lombardy!*; Lombardy region is administrated by a coalition of center-right and right wing parties.), ranking 2nd, and “#fermiamoloinsieme”, ranking 10th. What is, nevertheless, astonishing, is the presence, among the most used hashtags in all communities, of the pair [politician/TV program] (as “#mattino5”, “#lavitaindiretta”, “#ctcf”, “#dimartedì”). as if the main usage of hashtags is to promote the appearance of politicians in TV programs. Finally, hashtags by FI-L-FdI are mainly used to criticise the actions of the government, e.g., “#contedimettiti” (*Conte, resign!*).

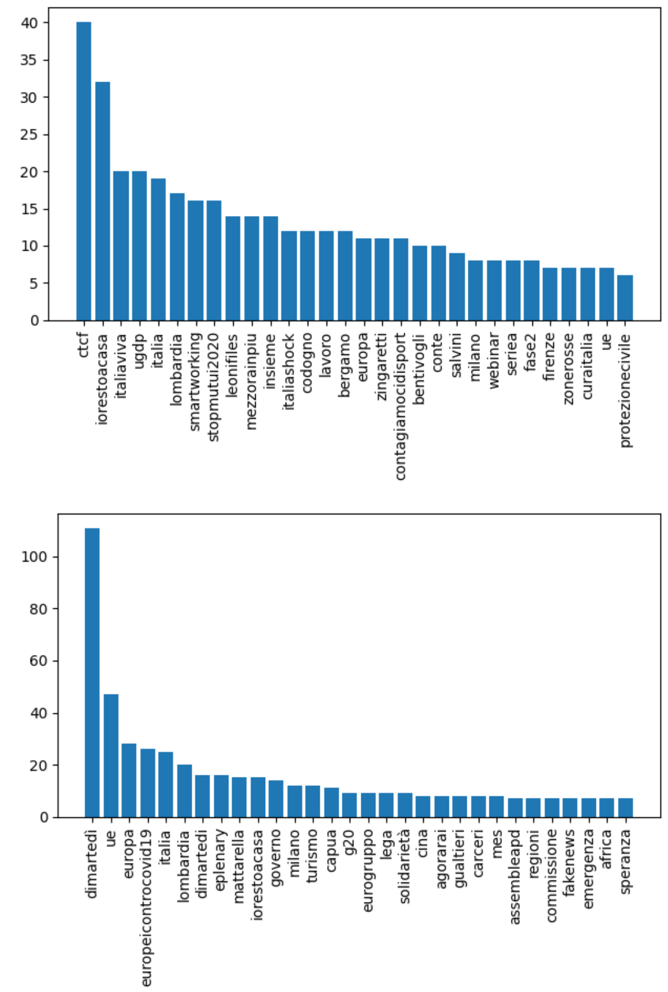

Figure 3 The 30 most diffused hashtags in the political sub-communities. Verified users. Top panel: Italia Viva (center-left); Bottom panel: Democratic Party (center-left).

4 Domain analysis for the directed validated network

Table 5 shows the number of tweets and retweets containing a url, and the tag assigned to the corresponding domain, for the directed validated network.

4.1 Hashtags by validated users

Sub-community	#url	R	∼R	NR	S	F	M	P	IS	ST	SE	UNC
FI-L-FdI	457746	38.3	12.1	22.1	4.7	0.1	0.0	0.3	0.0	0.0	0.3	22.1
Italia Viva	155125	58.7	6.7	0.7	3.6	0.5	0.1	0.6	0.6	0.0	0.0	28.5
Movimento 5 Stelle	120244	63.8	1.4	3.1	4.7	0.2	0.0	2.8	0.1	0.1	0.0	23.8
Partito Democratico	6183	47.5	1.5	0.4	5.9	1.0	0.0	0.1	2.9	0.0	0.0	40.7
`only tweets`												
FI-L-FdI	95902	29.5	9.6	30.6	4.7	0.2	0.0	0.2	0.0	0.0	0.0	25.2
Italia Viva	33648	47.8	14.4	1.1	2.9	0.5	0.0	0.5	0.0	0.0	0.0	32.8
Movimento 5 Stelle	22940	56.3	1.4	2.7	3.9	0.5	0.0	1.4	0.0	0.1	0.0	33.7
Partito Democratico	1759	35.6	0.9	0.2	3.5	0.8	0.0	0.0	1.2	0.0	0.0	57.8
`only retweets`												
FI-L-FdI	361844	40.7	12.8	19.9	4.8	0.1	0.0	0.4	0.0	0.0	0.4	20.9
Italia Viva	121477	61.8	4.6	0.6	3.7	0.5	0.1	0.7	0.7	0.0	0.0	27.3
Movimento 5 Stelle	97304	65.5	1.4	3.2	4.9	0.2	0.0	3.2	0.1	0.1	0.0	21.4
Partito Democratico	4424	52.2	1.7	0.5	6.8	1.0	0.0	0.1	3.6	0.0	0.0	34.1

Table 5 Domains annotation per political sub-communities – directed validated network. The impact of urls coming from Social Networks (S) is much lower than that in Table 4, when only verified users are considered. The consideration written in the caption of Table 4, about the high values of NR domains when considering only retweets, is valid here for M5S and PD only.

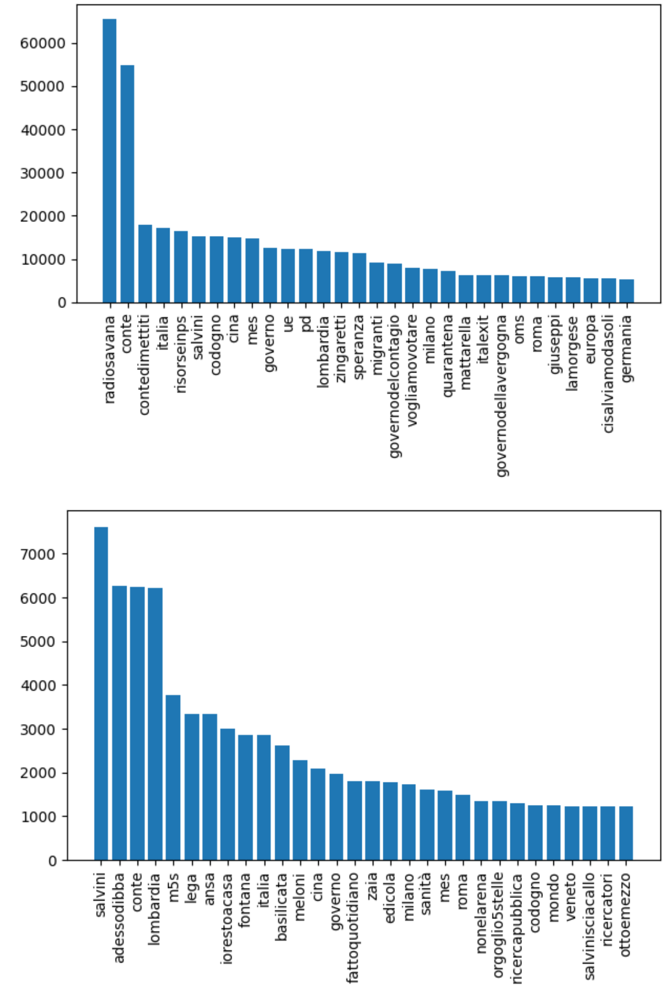

Figure 4 The 30 most diffused hashtags in the political sub-communities, directed validated network. Top panel: right and center-right wing discursive community. Bottom panel: 5 Stars Movement.

Figures 4 and 5 show the top 30 shared hashtags, for the various political subcommunities: the scales are different, due to the different activity of the various groups. Nevertheless, it is interesting to consider the most used hashtags in the various subcommunities in order to have an idea of the standings of the different parties. The opposition, represented by FI-L-FdI, shows dissatisfaction by using hashtags like '#contedimettiti' (*Conte, resign!*), '#governodellavergogna' (*government of the disgrace*), '#governodelcontagio' (*government of the contagion*) and '#vogliamovotare' (*we want to vote*).
Actually, the political competition still shines through the hashtags even for the other communities: it is the case, for instance, of Italia Viva. In the top 30 hashtags, we can find '#salvini', '#lega', but also '#papeete'[17], '#salvinisciacallo' (*Salvini jackal*) and '#salvinimmmerda' (*Salvini asshole*). Italia Viva use hashtags supporting the population: '#iorestoacasa', '#restoacasa' (*I am staying home*), '#restiamoacasa' (*let's stay home*). Criticisms towards the management of Lombardy health system during the pandemic can be deduced from the hashtag '#commissariatelalombardia' (*put Lombardy under receivership*) and '#fontana' (the Lega administrator of the Lombardy region).
Movimento 5 Stelle has the name of the main leader of the opposition, '#salvini', as first hashtag and it supports criticism to the Lombardy Administration with the hashtags '#fontanadimettiti' (*Fontana, resign!*) and '#gallera', the Health and Welfare Minister of the Lombardy Region, considered the main responsible for the bad management of the pandemic. Nevertheless, we can highlight even some hashtags encouraging the population during the lockdown, as the above mentioned '#iorestoacasa', '#restoacasa' and '#restiamoacasa'. It is worth mentioning that the government measures, and the corresponding M5S campaigns, are accompanied by specific hashtags: '#curaitalia' is the name of one of the decree of the prime Minister to inject liquidity in the Italian economy, '#acquistaitaliano' (*buy Italian products!*), instead, advertises Italian products to support the national economy.

5 Label propagation comparison

In the main text, we solved the problem of assigning the orientation to all relevant users in the validated retweet network via a label propagation. The approach is similar, but different to the one proposed in [3], the differences being in the starting labels, in the label propagation algorithm and in the network used. In this section we will revise the method employed in the present article, as compared it to the one in [3] and evaluate the deviations from other approaches.
First step of our methodology is to extract the polarisation of verified users from the bipartite network, as described in Section 5.1 of the main text, in order to use it as seed labels in the label propagation.

[17]Matteo Salvini, while Minister of Internal Affairs, prepared the political crisis in 2019 from the *Papeete Beach* resort in Milano Marittima, Italy (Il Sole 24Ore, *Salvini, dal Papeete all'opposizione: l'agosto terribile del "capitano"*, 1st September 2019). His staying was advertised by a huge TV and social media covering and marked as a lack of respect towards the Republican institutions by his opponents. Instead, his supporters admired his closeness to the population.

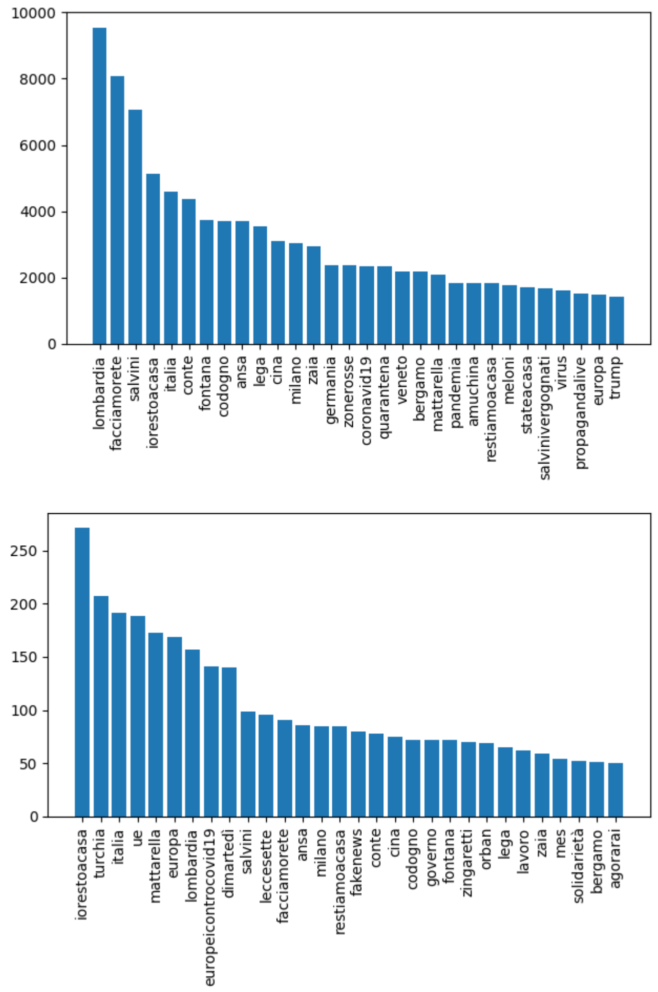

Figure 5 The 30 most diffused hashtags in the political sub-communities, directed validated network. Top panel: Italia Viva, Bottom panel: Democratic Party.

In reference [3], a measure of the "adherence" of the *unverified* users towards the various communities of *verified* users was used in order to infer their orientation, following the approach in [2], in turn based on the polarisation index defined in [5]. This approach was extremely performing when practically all unverified users interact at least once with verified one, as in [2]. While still having good performances in a different dataset as the one studied in [3], we observed isolated deviations: it was the case of users with frequent interactions with other unverified accounts of the same (political) orientation, randomly retweeting a different discursive community verified user. In this case, focusing just on the interaction with verified accounts, those nodes were assigned a wrong group. The labels for the polarisation of the unverified users defined [3] were subsequently used as seed labels in the label propagation. Due to the possibility described above of wrongly assigning labels to unverified accounts, in the present paper, we consider only the tags of verified users, since they pass a strict validation procedure and are more stable.

There is another difference in the label propagation used here against the one in [3]: in the present paper we used the label propagation of [6], while the one in [3] was quite home-made. As in reference [6], the seed labels of [3] are fixed, i.e. are not allowed to change[18]. The main difference is that, in case of a draw, among the labels of the first neighbours, in [6] a tie is removed randomly, while in the algorithm of [3] the label is not assigned and goes into a new run, with the newly assigned labels. Moreover, the updated of labels in [6] is asynchronous, while it is synchronous in [3]. We opted for the one in [6] for being actually a standard in the label propagation algorithms, being stable, more studied, and faster[19].
Finally, differently from the procedure in [3], we applied the label propagation not to the entire (undirected version of the) retweet network, but on the (undirected version of the) validated one. (The intent of choosing the *undirected version* is that in both case in which a generic account is significantly retweeting or being retweeted by another one, they do probably share some vision of the phenomena under analysis, thus we are not interested in the direction of the links, in this situation.) The rationale in using the validated network is to reduce the calculation time (due to the dimensions of the dataset), while obtaining an accurate result. While the previous differences from the procedure of [3] are dictated by conservativeness (the choice of the seed labels) or by the adherence to a standard (the choice of [6]), this last one may be debatable: why choosing the validated network should return "better" results than the ones calculated on the entire retweet network? We consider the case of a single day (in order to reduce the calculation time) and studied 6 different approaches:

1 a Louvain community detection [7] on the undirected version of the validated network of retweets;
2 a Louvain community detection on the undirected version of the unweighted retweet network;
3 a Louvain community detection on the undirected version of the weighted retweet network, in which the weights are the number of retweets from user to user;
4 a label propagation a la Raghavan et al. [6] on the directed validated network of retweets;
5 a label propagation a la Raghavan et al. on the unweighted retweet network;
6 a label propagation a la Raghavan et al. on the weighted retweet network, the weights being the number of retweets from user to user.

Actually, due to the order dependence of Louvain [8], we run several times the Louvain algorithm after reshuffling the order of the nodes, taking the partition in communities that maximise the modularity. Similarly, the label propagation of [6] has a certain level of randomness: we run it several times and choose the most frequent label assignment for every node.

[18]Actually, in [6] seed labels may be allowed to vary. Due to our application, we consider here the version in which they remain fixed, since the validation procedure is quite strict.
[19]In the present paper we used the implementation of the label propagation in [6] that can be found in the python-igraph python module.

In order to compare the results obtained with the various approaches, we calculated the Variation of Information (VI, [9]). VI considers exactly the different in information contents captured by two different partition, as consider by the Shannon entropy. Results are reported in the matrix in Figure 6 for the 23th of February (results are similar for other days). Even when using the weighted retweet network as "exact" result, the partition found by the label propagation of our approach has a little loss of information, comparable with the one of using an unweighted approach. Indeed, the results found by the various community detection algorithms show little agreement with the label propagation ones. Nevertheless, we still prefer the label propagation procedure, since the validated projection on the layer of verified users is theoretically sound and has a non trivial interpretation.

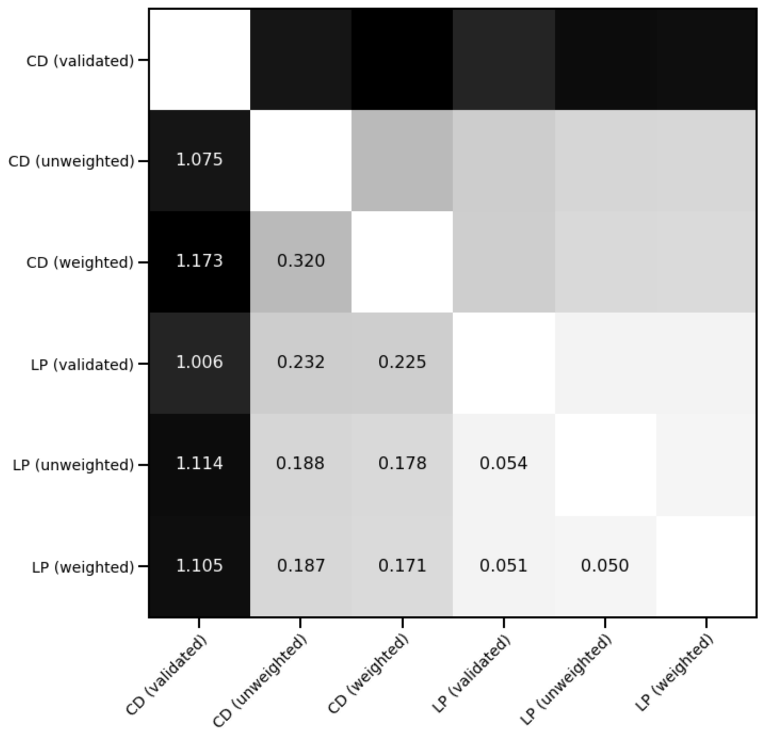

Figure 6 The Variation of Information table for the 23rd February 2020. (The date was chosen randomly.) The community detection algorithms do not agree so much even among themselves. Instead, the label propagation approaches results are quite similar. Due to this behaviour, we focus on the lightest one, i.e. the one calculated on the validated retweet network.

6 Fact-checking and low reputable news detection

The main result of this work quantifies the level of diffusion on Twitter of news published by sources considered scarcely reputable. Academy, Governments, and News Agencies are working hard to classify information sources according to criteria of credibility and transparency of published news. This is the case, for example, of NewsGuard, which we used for the tagging of the most frequent domains in the validated network of verified users and in the directed validated

network. As introduced in Subsection 4.2 of the main text, the NewsGuard browser extension and mobile app[20] offers a reliability result for the most popular news sources in the world, summarizing with a numerical score the level of their credibility and journalistic transparency.
With the same philosophy, but oriented towards US politics, the fact-checking site PolitiFact.com reports, with a 'truth meter', the degree of truthfulness of original claims made by politicians, candidates, their staffs, and, more, in general, protagonists of US politics. One of the eldest fact-checking websites dates back to 1994: in addition to political figures, snopes.com is a fact-checker for hoaxes and urban legends.
Generally speaking, a fact-checking site has behind it a multitude of editors and journalists who, with a great deal of energy, manually check the reliability of a news, or of the publisher of that news, by evaluating criteria such as, e.g., the tendency to correct errors, the nature of the newspaper's finances, and if there is a clear differentiation between opinions and facts. Recent attempts tried to automatically find articles worthy of being fact-checked. For example, work in [10] uses a supervised classifier, based on an ensemble of neural networks and Support Vector Machines, to figure out which politician claims need to be debunked, and which have already been debunked.
Despite the tremendous effort of stakeholders to keep the fact-checking sites up to date and functioning, disinformation may resist debunking due to a combination of factors. There are psychological aspects, like the quest for belonging to a community and getting reassuring answers, the adherence to one's viewpoint, a native reluctance to change opinion [11, 12], the formation of echo chambers [13], where people polarize their opinions as they are insulated from contrary perspectives. These are key factors for people to contribute to the success of disinformation spreading [14, 15]. Moreover, researchers demonstrated how the spreading of false news is strategically supported by the massive and organized use of trolls and bots [16].
In spite of the need to educate the user to a conscious fruition of online information through means also different from those represented by technological solutions, there are a series of promising works that exploits classifiers to tag a news as credible or not.
One interesting approach is based on the analysis of spreading patterns on social platforms. Monti et al. recently provide a deep learning framework for detection of fake news cascades [17]. A ground truth is acquired by following the example by Vosoughi et al. [18], collecting Twitter cascades of verified false and true rumors. Employing a novel deep learning paradigm for graph-based structures, cascades are classified based on user profile, user activity, network and spreading, and content. The main result of the work is that 'a few hours of propagation are sufficient to distinguish false news from true news with high accuracy'. This result has been confirmed by other studies too. Work in [19], by Zhao et al., examines diffusion cascades on Weibo and Twitter: focusing on topological properties, such as the number of hops from the source and the heterogeneity of the network, the authors demonstrate that networks in which fake news are diffused feature

[20]https://www.newsguardtech.com/

characteristics really different from those diffusing genuine information. Diffusion networks appear a definitive path to follow for fake news detection. This is also confirmed by Pierri et al. [20]: also there, the goal is to classifying news articles pertaining to bad and genuine information, 'by solely inspecting their diffusion mechanisms on Twitter'. Even in this case, results are impressive: a simple Logistic Regression model is able to correctly classify news articles with a very high accuracy (AUROC up to 94%).

7 Newsguard in a nutshell

This section gives more details about Newsguard and the criteria adopted by its developers to tag news sources. Much of the content here is inherited from the website
https://www.newsguardtech.com/ratings/rating-process-criteria/, lastly accessed on April 29, 2021.
NewsGuard employs a team of trained journalists and experienced editors to review and rate news websites based on nine journalistic criteria. The criteria assess basic practices of credibility and transparency. Based on a site's performance on these nine criteria, it is assigned a red or green rating, indicating its credibility. Each criterion is worth a certain number of points out of 100. A site with a score of 60 points or higher receives a green rating. A site with a score lower than 60 points receives a red rating.

Credibility Criteria:

- **Does not repeatedly publish false content**: The site does not repeatedly produce stories that have been found—either by journalists at NewsGuard or elsewhere—to be clearly and significantly false, and which have not been quickly and prominently corrected. (22 Points)
- **Gathers and presents information responsibly**: Content providers are generally fair and accurate in reporting and presenting information. They reference multiple sources, preferably those that present direct, firsthand information on a subject or event or from credible second hand news sources, and they do not egregiously distort or misrepresent information to make an argument or report on a subject. (18 Points)
- **Regularly corrects or clarifies errors**: The site makes clear how to report an error or complaint, has effective practices for publishing clarifications and corrections, and notes corrections in a transparent way. (12.5 Points)
- **Handles the difference between news and opinion responsibly**: Content providers who convey the impression that they report news or a mix of news and opinion distinguish opinion from news reporting, and when reporting news, do not egregiously cherry pick facts or stories to advance opinions. Content providers who advance a particular point of view disclose that point of view. (12.5 Points)
- **Avoids deceptive headlines**: The site generally does not publish headlines that include false information, significantly sensationalize, or otherwise do not reflect what is actually in the story. (10 Points)

Transparency Criteria:

- **Website discloses ownership and financing**: The site discloses its ownership and/or financing, as well as any notable ideological or political positions held by those with a significant financial interest in the site, in a user-friendly manner. (7.5 Points)
- **Clearly labels advertising**: The site makes clear which content is paid for and which is not. (7.5 Points)
- **Reveals who's in charge, including possible conflicts of interest**: Information about those in charge of the content is made accessible on the site. (5 Points)
- **The site provides the names of content creators, along with either contact or biographical information**: Information about those producing the content is made accessible on the site. (5 Points)

The process of assigning a rating against the criteria listed above is as follows: A NewsGuard analyst assesses the contents of the site against the nine criteria. The analyst drafts a written 'Nutrition Label' for the site based on their reporting. Nutrition labels consist of a grid showing the site's performance on each of the nine criteria and a written explanation of the content on the site, who's behind it, and why it received its rating. NewsGuard calls a website that fails one or more criteria for comment. If the website provides a comment, that comment is included in the written assessment of the site to provide users with the website's perspective. The rating is reviewed and fact-checked by experienced editors. The Nutrition Labels are periodically updated.

Ratings: Here, we report the ratings attached to the information source after the evaluation of the nine criteria on that source.

- **Green**: A website is rated green if it generally adheres to basic standards of credibility and transparency. Significant failures in satisfying one or more criteria are reported in the evaluation, with related explanations.
- **Red**: A website is rated red if it generally fails to meet basic standards of credibility and transparency. Severe violations to journalistic standards are reported in the evaluations.
- **Satire**: A humor or satire site is tagged as not a real news website. Newsguard does not rate these sites according to the nine journalistic criteria, but provides a description of each site including, if possible, who is behind it.
- **Platform**: A platform site indicates a site that primarily hosts user-generated content that it does not vet. Newsguard provides a description of each site and its practices.

Author details

[1] Department of Molecular Sciences and Nanosystems, Ed. Alfa, Via Torino 155, 30170 Venezia Mestre, Italy. [2] European Centre for Living Technology (ECLT), Ca' Bottacin, 3911 Dorsoduro Calle Crosera, 30123 Venice, Italy. [3]IMT Alti Studi Lucca, Piazza San Francesco 19, 55100 Lucca, Italy. [4]Institute of Informatics and Telematics, National Research Council, via Moruzzi 1, 56124, Pisa, Italy.

References

1. M. Nacoti, A. Ciocca, A. Giupponi, P. Brambillasca, F. Lussana, M. Pisano, G. Goisis, D. Bonacina, F. Fazzi, R. Naspro, L. Longhi, M. Cereda, C. Montaguti, At the Epicenter of the Covid-19 Pandemic and Humanitarian Crises in Italy: Changing Perspectives on Preparation and Mitigation, Catal. non-issue content (2020).

2. C. Becatti, G. Caldarelli, R. Lambiotte, F. Saracco, Extracting significant signal of news consumption from social networks: the case of Twitter in Italian political elections, Palgrave Commun. (2019)
3. G. Caldarelli, R. De Nicola, F. Del Vigna, M. Petrocchi, F. Saracco, The role of bot squads in the political propaganda on Twitter, Commun. Phys. **3**(1), 1 (2020).
4. T. Radicioni, E. Pavan, T. Squartini, F. Saracco, Analysing Twitter Semantic Networks: the case of 2018 Italian Elections, arXiv (2020). URL http://arxiv.org/abs/2009.02960
5. A. Bessi, F. Zollo, M. Del Vicario, M. Puliga, A. Scala, G. Caldarelli, B. Uzzi, W. Quattrociocchi, Users polarization on Facebook and Youtube, PLoS One **11**(8) (2016).
6. U.N. Raghavan, R. Albert, S. Kumara, Near linear time algorithm to detect community structures in large-scale networks, Phys. Rev. E - Stat. Nonlinear, Soft Matter Phys. (2007).
7. V.D. Blondel, J.L. Guillaume, R. Lambiotte, E. Lefebvre, Fast unfolding of communities in large networks, J. Stat. Mech. Theory Exp. **10008**(10), 6 (2008).
8. S. Fortunato, M. Barthélemy, Resolution limit in community detection., Pnas **104**(1), 36 (2007). . URL http://www.ncbi.nlm.nih.gov/pubmed/17190818http://www.pubmedcentral.nih.gov/articlerender.fcgi?artid=PMC1765466
9. M. Meila, Comparing clusterings by the variation of information, Learn. theory Kernel Mach. 16th Annu. Conf. Learn. Theory 7th Kernel Work. COLT/Kernel 2003, Washington, DC, USA, August 24-27, 2003 Proc. p. 173 (2003).
10. P. Atanasova, P. Nakov, L. Màrquez, A. Barrón-Cedeño, G. Karadzhov, T. Mihaylova, M. Mohtarami, J.R. Glass, Automatic fact-checking using context and discourse information, J. Data and Information Quality **11**(3), 12:1 (2019)
11. G. Walton, G. Cohen, A question of belonging: Race, social fit, and achievement, Journal of Personality and Social Psychology pp. 82–96 (2007)
12. D. Webster, A. Kruglanski, Cognitive and social consequences of the need for cognitive closure, European Review of Social Psychology **8**(1), 133 (1997).
13. M. Del Vicario, G. Vivaldo, A. Bessi, F. Zollo, A. Scala, G. Caldarelli, W. Quattrociocchi, Echo Chambers: Emotional Contagion and Group Polarization on Facebook, Sci. Rep. (2016).
14. D. Calvert. The psychology behind fake news (2017)
15. J. De keersmaecker, A. Roets, Fake news: Incorrect, but hard to correct. the role of cognitive ability on the impact of false information on social impressions, Intelligence **65**, 107 (2017).
16. C. Shao, G.L. Ciampaglia, O. Varol, K.C. Yang, A. Flammini, F. Menczer, The spread of low-credibility content by social bots, Nat. Commun. **9** (2018)
17. F. Monti, F. Frasca, D. Eynard, D. Mannion, M.M. Bronstein, Fake news detection on social media using geometric deep learning, CoRR **abs/1902.06673** (2019). URL http://arxiv.org/abs/1902.06673
18. S. Vosoughi, D. Roy, S. Aral, The spread of true and false news online, Science **359**(6380), 1146 (2018).
19. Z. Zhao, J. Zhao, Y. Sano, O. levy, H. Takayasu, M. Takayasu, D. Li, J. Wu, S. Havlin. Fake news propagate differently from real news even at early stages of spreading (2018)
20. F. Pierri, C. Piccardi, S. Ceri, A multi-layer approach to disinformation detection on twitter, CoRR **abs/2002.12612** (2020). URL https://arxiv.org/abs/2002.12612
